# Combining laser capture microdissection and proteomics reveals an active translation machinery controlling invadosome formation

**DOI:** 10.1038/s41467-018-04461-9

**Published:** 2018-05-23

**Authors:** Zakaria Ezzoukhry, Elodie Henriet, Fabrice P. Cordelières, Jean-William Dupuy, Marlène Maître, Nathan Gay, Sylvaine Di-Tommaso, Luc Mercier, Jacky G. Goetz, Marion Peter, Frédéric Bard, Violaine Moreau, Anne-Aurélie Raymond, Frédéric Saltel

**Affiliations:** 1 INSERM, UMR1053, BaRITOn Bordeaux Research in Translational Oncology, 146 Rue Léo Saignat, Bordeaux, F-33000 France; 2 Université de Bordeaux, 146 Rue Léo Saignat, Bordeaux, F-33076 France; 3 Bordeaux Imaging Center, UMS 3420 CNRS-Université de Bordeaux-US4 INSERM, Pôle d’imagerie photonique, 146 Rue Léo Saignat, Bordeaux, F-33000 France; 4 Plateforme Protéome, Centre de Génomique Fonctionnelle, 146 Rue Léo Saignat, Bordeaux, F-33000 France; 5 Neurocentre Magendie U1215, INSERM, 146 Rue Léo Saignat, Bordeaux, F-33000 France; 6 Inserm U1109 MN3T, 1 Place de L’Hôpital, Strasbourg, F-67000 France; 7 LabEx Medalis, Université de Strasbourg, Strasbourg, F-67000 France; 8 Fédération de Médecine Translationnelle de Strasbourg (FMTS), Strasbourg, F-67000 France; 9 IGMM, CNRS, Univ. Montpellier, 1919 Route de Mende, Montpellier, F-34090 France; 10 Institute of Molecular and Cell Biology, 61 Biopolis Drive, Proteos, Singapore; 11 Oncoprot, INSERM UMR1053-TBM Core US005, 146 Rue Léo Saignat, Bordeaux, F-33000 France; 12 Present Address: Mohammed VI University of Health Sciences (UM6SS), Casablanca, Morocco

## Abstract

Invadosomes are F-actin-based structures involved in extracellular matrix degradation, cell invasion, and metastasis formation. Analyzing their proteome is crucial to decipher their molecular composition, to understand their mechanisms, and to find specific elements to target them. However, the specific analysis of invadosomes is challenging, because it is difficult to maintain their integrity during isolation. In addition, classical purification methods often suffer from contaminations, which may impair data validation. To ensure the specific identification of invadosome components, we here develop a method that combines laser microdissection and mass spectrometry, enabling the analysis of subcellular structures in their native state based on low amounts of input material. Using this combinatorial method, we show that invadosomes contain specific components of the translational machinery, in addition to known marker proteins. Moreover, functional validation reveals that protein translation activity is an inherent property of invadosomes, which is required to maintain invadosome structure and activity.

## Abstract (web summary)

Invadosomes degrade extracellular matrix and facilitate cell invasion but their molecular composition is not fully understood. Here, the authors combine laser capture and mass spectrometry to map the proteome of invadosomes, showing that they rely on internal translational activity to maintain their structure.

## Introduction

Invadosomes is a collective term for podosomes and invadopodia observed respectively in normal and cancer cells^1,2^. They consist of dynamic F-actin structures involved in different functions such as adhesion, mechano-transduction, and signaling. The specific feature of invadosomes is their capacity to degrade extracellular matrix. Invadosomes exist in different forms depending on the cell type and the cellular microenvironment. Indeed, growth factors, cytokine stimulation, composition, and organization of the extracellular matrix can all modulate invadosome formation and organization, either as individual dots, aggregates, rosettes, or linear invadosomes^3,4^. Depending on the cell type, the matrix degradation activity is associated with various cellular functions such as angiogenesis for endothelial cells or bone resorption for osteoclasts^1^. Invadosomes were also described in vivo and their presence in cancer cells is correlated with invasiveness^5,6^. Hence, it is crucial to determine their molecular composition to investigate their modus operandi.

The real challenge with invadosomes is the difficulty in purifying these structures. Indeed, invadosomes are dynamic F-actin structures that share common components with other actin structures in cells such as lamellipodia, filopodia, stress fibers, and membrane ruffles. For example, focal adhesions associated with actin stress fibers share common molecular elements with invadosomes such as talin, vinculin, and paxillin. Several studies have centered on the focal adhesion proteome^7–9^. By contrast, only a few studies, which relied on conventional differential cell lysis or subcellular fractionation with their well-known limitations, attempted to elucidate the invadosome protein composition^10–13^.

More generally, the identification of proteins forming subcellular complexes not only improves our understanding of their functions but also the cellular mechanisms. Currently, the combination of mass spectrometry (MS)-based proteomics with biochemical fractionation or immunoprecipitation is the classical approach for the characterization of protein interactions in subcellular complexes^14,15^. Typically, mechanically prepared cell homogenates contain a mixture of various organelles or cellular compartments, such as cytoplasmic membranes and cytoskeletal portions, which can be fractionated by centrifugation and/or density gradient centrifugation^15^. Isolation of specific subcellular organelles, structures, or protein complexes is particularly challenging due to the mechanical cellular lysis that disrupts them directly. For example, adhesive structures (focal adhesions or invadosomes), cell–cell junctions or cytoskeleton structures (filopodia, stress fibers, lamellipodia, pseudopodia) are disassembled during cell lysis. Various strategies were developed to conserve the integrity of these subcellular organizations, as performed for pseudopodia^16,17^. However, difficulties still persist to isolate them specifically^8,18^.

Previous studies used a combination of laser capture microdissection and MS analysis for the molecular characterization of specifically isolated cells or tissue sections but these approaches were not applied at the subcellular level^19–21^. In this study, we develop a method that combines laser capture microdissection and MS to map the invadosome proteome on fixed cells. We present a strategy, based on structure tracking as previously described for pseudopodia, lamellipodia, or invadopodia^22–24^, to automate laser capture and greatly facilitate the collection of invadosomes. Owing to the sensitivity of the latest generation of mass spectrometers, these small amounts of material can then be analyzed by MS-based proteomics. To guarantee the specificity of the identified proteins, we combine the proteomics analysis with isotopic labeling, accounting for the fact that the high sensitivity mass spectrometric analysis may otherwise result in the identification of undesirable contaminating proteins^25^.

In this study, our main goal is to minimize contamination of other actin structures while maintaining the cell and invadosome integrity. After analyzing the proteome of microdissected and collected invadosomes, we confirm the enrichment of identified proteins by label-free quantification, comparing invadosome protein abundances against a total cell lysate. We show that a large number of the enriched proteins is involved in messenger RNA translation. Following validation of their presence in invadosomes, we establish here that invadosomes concentrate mRNA and exhibit their own translational activity.

## Results

### Combining laser capture and proteomics to study invadosomes

**Fig. 1:**
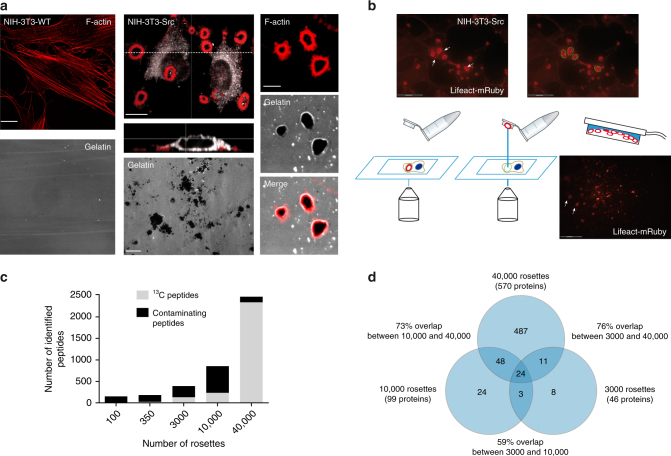
Laser capture and collection of rosettes, global identification values and reproducibility of identification. **a** Left panel: representative confocal images of NIH-3T3 wild-type (WT) cells presenting only stress fibers when seeded on a fluorescent gelatin matrix (as seen in the image below). **a** Middle panel: representative confocal images of NIH-3T3-Src cells with invadosome rosettes revealed with the Lifeact-mRuby (red). Orthogonal view section shows the thickness of those structures. Below, a representative image of an in situ zymography assay shows the degradation capacity of the cells when seeded on a fluorescent gelatin matrix. Scale bars: 10 µm. **a** Right panel: representative confocal images of rosettes degrading the fluorescent gelatin matrix. Scale bars: 10 µm. **b** Upper panel: representative confocal images of NIH-3T3-Src cells with rosettes. In the right image, the dotted circles surround the rosettes that were microdissected (scale bar: 30 µm); **b** lower panel: schematic representation of the microdissection process. The laser cuts the region of interest, which will then be propelled into the cap of a tube. The last panel shows microdissected rosettes (Lifeact-mRuby, red) collected into the cap of the tube (scale bar: 300 µm). **c** Number of identified peptides according to the number of collected rosettes. Due to previous metabolic isotopic labelling, peptides coming from dissected invadosomes were identified with ^13^C modifications (^13^C peptides) and discriminated from external contamination (contaminating peptides). **d** Proteins identified in each experiment (increasing number of collected invadosomes) were compared with estimate the reproducibility of identification

For this study, we used NIH-3T3-Src cells, which are Src-transformed mouse fibroblasts, as an invadosome model^26^. We generated a NIH-3T3-Src cell line stably expressing mRuby-LifeAct to detect invadosomes without exogenous staining. These cells have the advantage of forming large rosettes (diameter up to 5–7 μm) and most often present several invadosomes in one cell, whereas NIH-3T3-WT cells form only stress fibers (Fig. 1a). The position of the invadosome rosettes was miscellaneous, some localized under the nucleus or near the central core of the cell or at the extremity of membrane protrusions (Fig. 1a). An orthogonal view of invadosome rosettes shows that these structures occupy a large part of the cell thickness (Fig. 1a). We confirmed matrix degradation activity associated with the invadosome rosettes using an in situ zymography assay with fluorescent gelatin only with NIH-3T3-Src cells (Fig. 1a). For our purpose, it is important to work with fixed cells due to the dynamics of these invadosome rosettes (see Supplementary Movie 1).

To discriminate laser captured proteins from undesirable exogenous contaminating proteins, NIH-3T3-Src cells were metabolically labeled using stable isotope labeling with amino acids in cell culture (SILAC) method (Supplementary Fig. 1a)^25,27^. This first step guarantees the specificity of identifications. mRuby-LifeAct staining of paraformaldehyde (PFA)-fixed cells was used to precisely visualize and select invadosomes to be microdissected.

Laser microdissection enabled invadosome isolation, minimizing contaminations from other cellular elements. Performing microdissection involved manual delineation of invadosomes. A laser beam was used to cut the selected regions, and to propel and collect them into a tube cap (Fig. 1b). We imaged cells before and after microdissection showing that invadosome rosettes were indeed micro-dissected (Supplementary Fig. 2).

Isolating invadosomes for proteomic analysis involves dissecting a lot of structures to obtain enough material. We started experiments with a manual collection of 100 rosettes. Following protein extraction, fixation reversion, and protein digestion, the liquid cromatography-tandem MS (LC-MS/MS) analysis identified only two proteins with five ^13^C peptides. As one of these proteins was actin, we were reassured about our approach but concluded that we did not collect enough material. Therefore, we increased the number of manually collected rosettes and, as expected, identified more ^13^C proteins each time (Fig. 1c).

Once we manually collected 10,000 rosettes, the task of microdissection became too difficult and time consuming. To speed up the process, we have developed a strategy that benefits from ImageJ software’s automated processing. As a first step, under the Zeiss PALM software, the user located a field of interest. An image was taken, which was automatically imported under the ImageJ software (Supplementary Fig. 1b). Using a homemade plugin, metadata were extracted. This information allowed accessing the precise acquisition stage location, expressed as a calibrated set of coordinates. A homemade ImageJ toolset used those coordinates to calculate a matrix of points placed at the center of adjacent field. Using the plugin, this matrix was exported as an “Element file”, a proprietary file format from Zeiss, and manually imported into the PALM software. The microdissection system was then operated to move the stage at each of the coordinates, and to acquire an image for each visited field. The dissection process was finally completed based on the automatically segmented regions (Supplementary Fig. 1b). When performed semi-automatically, an experienced person could dissect on an average 900 structures per hour, which is a fourfold improvement on the manual procedure. Moreover, after automatic selection of the regions of interest (ROI), the operator can control the selection. Indeed, the operator can deselect the aberrant and not specific structures to improve the quality control of the process. This point can affect the throughput of the method. However, using pictures extracted from the microdissection process, we can evaluate that the criteria used to select invadosomes are very strict. Thus, the automation system allowed us to save time and collect 40,000 rosettes more comfortably.

Over the experiments, a database search analysis with ^13^C(6) K or ^13^C(6) R labeling as variable modification showed that a large majority (67–97%) of identified peptides in the first experiments were not ^13^C labeled and thus came from contaminations. The amount of contaminating proteins decreased with more cellular material and became a minority (5% for 40,000 rosettes collected) as soon as we exceeded the sensitivity threshold of the mass spectrometer (Fig. 1c). This result demonstrates the relevance of isotopic labeling prior to laser microdissection to guarantee the specificity of protein identifications.

**Fig. 2:**
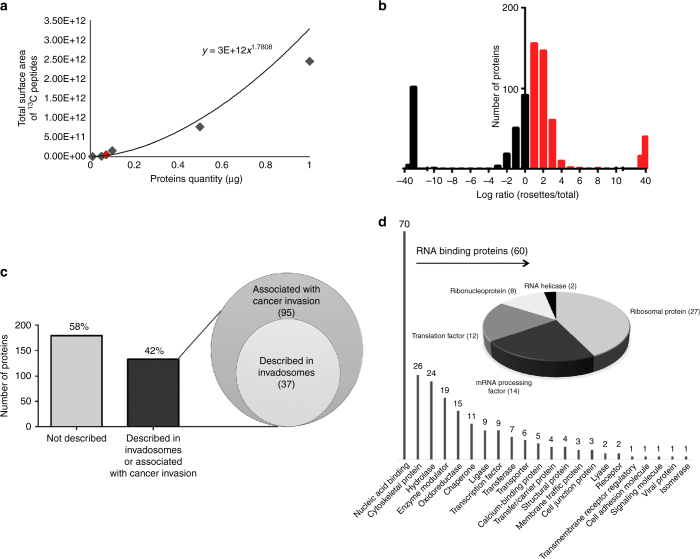
Enrichment analysis and functional classification of proteins. **a** The closest point of the total protein quantity range (100 ng) was chosen to compare the MS relative abundances after normalisation. **b** Among the 366 proteins identified with at least 2 peptides in the rosettes sample, 312 proteins were enriched with a rosette/total proteome abundance ratio ≥ 1.5 (log ratio ≥ 0.6 in red). **c** Manual sorting of the proteins according to their characterization in the literature. Among the 312 enriched proteins, 42% (95 proteins) were associated to cancer invasion including 37 proteins already described in the invadosomes. **d** The bart chart represents the classification of the 312 enriched proteins according to the GO categories (“Protein class” classification from the PANTHER database). Among the 70 nucleic acid proteins, 60 proteins were sub-classified as RNA-binding proteins whose functional assignments distribution is represented into the pie chart

With 40,000 isolated rosettes, we have identified 2286 ^13^C peptides corresponding to 570 proteins with at least one specific peptide or 366 proteins with at least 2 specific peptides (Fig. 1c and Fig. 2a). We compared the proteins identified in each experiment and found an overlap of 59–76% (Fig. 1d), suggesting a good reproducibility of the entire process.

### Enrichment analysis and classification of invadosome proteins

To identify proteins that are enriched in invadosome rosette samples, we compared relative protein abundances between the microdissected fraction and the total extract of labeled cells using label-free quantification. We chose to use the total cell extract in order to avoid protein enrichment of another specific subcellular structure and to obtain average protein expression levels across of the proteome. With such small amount of collected material, we could not measure the protein concentration and therefore directly analyzed an equal amount of total extract proteins. We opted to estimate protein concentration with LC-MS/MS analysis using a dilution range of a total extract of ^13^C labeled NIH-3T3-Src cells. We used the sum of surface area of all detected ^13^C peptides as readout to evaluate the injected peptide quantities. We deduced 72 ng for the peptide quantity contained in the sample of 40,000 rosettes and chose the closest point of the total protein quantity range for quantification analysis (Fig. 2a and Supplementary Table 1). We then normalized MS abundances on the sum of detected peaks and performed a relative label-free quantification between proteins identified in the rosette sample and the whole proteome (Fig. 2b). Among the 366 proteins identified with at least 2 peptides in the rosette sample, 312 proteins were enriched with a rosette/total proteome abundance ratio ≥ 1.5, indicating the significant presence of these proteins in the rosettes (Fig. 2b, Supplementary Table 2, and Supplementary Data 1).

We then used Gene Ontology (GO) (26) to classify the identified invadosome proteins (Fig. 2d). Thirty-seven proteins, including Arp2/3 complex subunits, cortactin, talin and vimentin, were already described in invadosomes and, more widely, 131 proteins (42%) were associated with tumor invasion (Supplementary Table 3, Supplementary Data 2, and Fig. 2c), substantiating the relevance of our findings and providing opportunities to reveal key invadosome proteins not yet identified. With the aim of associating biological functions with identified proteins, we classified the 312 enriched proteins according to GO categories (Fig. 2d). As expected, we found an enrichment of cytoskeletal proteins (26 identifications). Interestingly, we also found a large proportion of nucleic acid-binding proteins (22%, 70 proteins) and, more precisely, RNA-binding proteins (19%, 60 proteins) (Fig. 2d).

Next, we generated a functional network of proteins identified and enriched in invadosomes compared with the whole cellular proteome (Supplementary Fig. 3). Annotations were attributed manually for the following functions: cancer invasion, invadosomes, and matrix degradation. Involvement in actin reorganization, cell adhesion, chemotaxis, or protein translation was extracted from the Ingenuity® Pathway Analysis Database (Qiagen) (Supplementary Fig. 3). According to this analysis, the two major emerging groups are cancer invasion and translation. The identification of several ribosomal proteins (27), ribonucleoproteins (8), mRNA processing factors (14), and translation factors (12) in the invadosome proteome (Fig. 2d) suggests that a dedicated protein synthesis machinery is associated with these structures.

### A translation machinery associated with invadosomes

The spatio-temporal control of protein translation is necessaryto ensure protein production at the right time and place^28^. This was already established in neurons and for focal adhesions^29,30^. Translational control is also crucial in cancer development, especially the selective control of the translation of specific mRNAs that promote tumor progression including invasion and metastasis^31^.

**Fig. 3:**
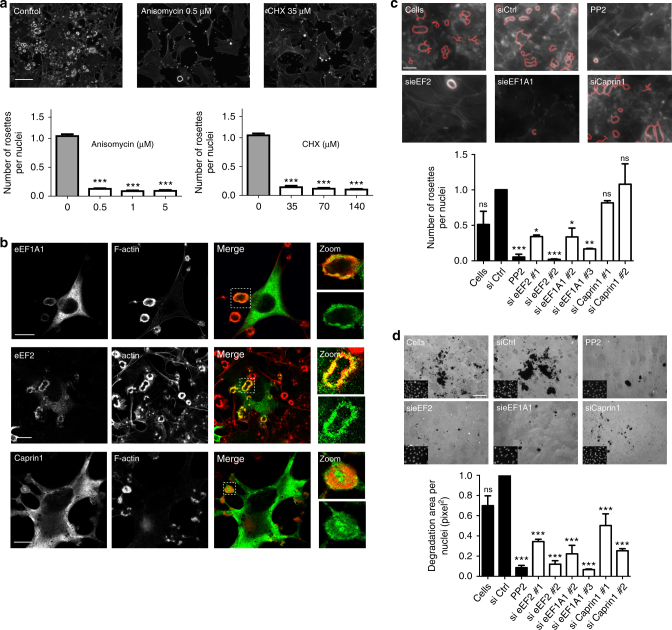
Localization and involvement of translation-related proteins at invadosomes. **a** Dose response of the number of rosettes per nuclei after translation inhibitor treatment. NIH-3T3-Src cells were treated with anisomycin or cycloheximide (CHX) for 24 h. Representatives images of cells treated the minimum concentration. The bar graph represents the number of rosettes per nuclei. Error bars (SEM, *n* = 20 fields, three independent experiments; ***P < 0.001 as compared with the non-treated cells as control, one-way ANOVA followed by Bonferroni test as compared with the siRNA control). Scale bar: 50 µm. **b** Confocal microscopy images of lifeact-mRuby (red)-expressing NIH-3T3-Src cells immunostained for eEF1A1 (green) or transfected with eEF2-GFP (green) or Caprin 1-myc (green). Left panels show in each channel in black and white. Right panels show merge images with enlarged views of the boxed regions. Scale bars: 10 µm (EEF1A1 and Caprin 1), 20 µm (eEF2). **c** Lifeact-mRuby (gray)-expressing NIH-3T3-Src cells were transfected with a siRNA control (siCtrl) or two independent siRNA targeting eEF2, eEF1A1, or Caprin 1 involved in translation activity. As controls the cells were treated with the Src inhibitor PP2 (5 µM). The upper panel shows representative images of the rosette number determined by the mask applied by the software (red areas). Scale bar: 5 µm. In the lower panel, the bar graph shows the number of rosettes per nuclei. The black bars represent the control conditions of the experiment. Error bars (SEM, *n* = 75 fields, three independent experiments; ns, not significant; *P < 0.05; **P < 0.005; ***P < 0.001 by one-way ANOVA followed by Bonferroni test as compared with the siRNA control). **d** NIH-3T3-Src cells transfected with a siRNA control (siCtrl) or two independent siRNA targeting eEF2, eEF1A1, or Caprin 1 were seeded on a fluorescent gelatin matrix. As a control, cells were treated with PP2 (5 µM). The upper panel shows representative images of the degraded area (black), insets on the bottom show the nuclei of the same field. Scale bar: 50 µm. In the lower panel, the bar graph shows the gelatin area degraded per cell after 24 h. Error bars (SEM, *n* = 30 fields, three independent experiments; ns, not significant, ***P < 0.001 as compared with the control siRNA, one-way ANOVA followed by Bonferroni test as compared to the siRNA control)

As our proteomics data suggest that invadosomes are enriched for proteins of the protein translational machinery, we first studied the impact of translation inhibitors in invadosome formation. Anisomycin and cycloheximide (CHX) treatment in concentration-range and time-course assays impacted invadosome formation in NIH-3T3-Src cells (Fig. 3a and Supplementary Fig. 4a). This effect seems to be specific to invadosomes compared with other F-actin structures such as stress fibers or lamellipodia^32,33^.

Then, we confirmed that the identified and enriched proteins from the translational machinery are indeed localized to invadosomes. Using an immunofluorescence approach, we demonstrated that caprin 1, eukaryotic elongation factor 2 (eEF2), eukaryotic translation elongation factor 1 ɑ1 (eEF1A1), and eukaryotic translation initiation factor 3 subunit H and L (eIF3H, eIF3L) colocalize with invadosomes (Fig. 3b and Supplementary Fig. 4d). Interestingly, the localization could be different depending on the protein tested. Indeed, eEF2, eEF1A1, eIF3H and eIF3L colocalized with the F-actin core, whereas caprin 1 concentrated in the center and at the periphery of the rosette. Moreover, a dynamic observation of eEF2 showed that the localization of this protein could evolve during the maturation process of the invadosome rosette (Supplementary Fig. 4b). We also analyzed the localization of these proteins in the presence of linear invadosomes, which are formed when NIH-3T3-Src cells are seeded into type I collagen fibrils^34^. Even though this different actin organization is associated with a modification of the molecular composition of invadosomes, they still colocalize with the translation-related proteins (Supplementary Fig. 5).

Furthermore, to address the involvement of identified proteins and to validate our methodology, we performed a small interfering RNA (siRNA) screen on 18 proteins chosen according to their enrichment ratio. Twenty-eight percent of the siRNAs tested and 36% of the siRNA targeting translation-related proteins had an impact on invadosome formation in our assay (Supplementary Fig. 4c). More precisely, depletion of eEF2 and eEF1A decreased invadosome formation; on the contrary, caprin 1 knockdown did not affect the number of invadosomes per nuclei (Fig. 3c). In parallel, we measured the degradation activity of the cells in the same conditions and observed a decrease in the matrix degradation capacity of the cells upon silencing of all three proteins (Fig. 3d). To validate our result, we tested the impact of the depletion of eEF2 and eEF1A on linear invadosomes in hepatoma HuH6 cells^4^. We demonstrated that their silencing altered both invadosome formation and associated-matrix degradation (Supplementary Fig. 6).

These data confirm the robustness of our method to identify the invadosome proteome and demonstrate a subcellular concentration of the translation machinery at invadosomes.

### Protein translation activity associated with invadosomes

**Fig. 4:**
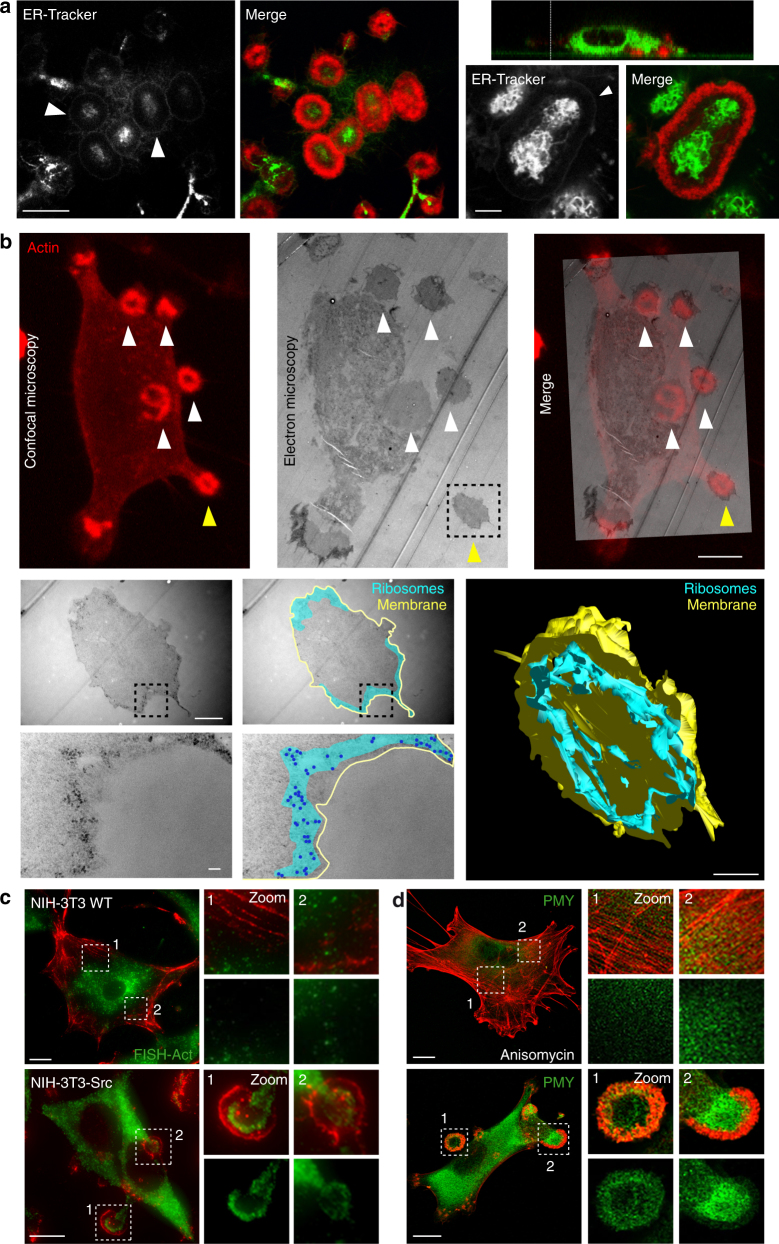
A protein translation activity associated with invadosome rosettes. **a** Lifeact-mRuby (red)-expressing NIH-3T3-Src cells were stained with an ER-tracker (green). Representative confocal images are shown. On the left panel, a *Z*-stack reconstruction shows an invagination of the ER into a invadosome rosette. A magnification of a rosette shows the ER organization into this structure. Scale bars: 10 µm (right panel) and 2 µm (left panel). **b** Upper panel: correlative light and electron microscopy (CLEM) in vitro. First, a confocal acquisition of a lifeact-mRuby (red)-expressing NIH-3T3-Src cell forming rosettes (arrow heads) is taken. Then, the exact same cell is imaged by transmitted electron microscopy (TEM). Next, the position of rosettes is correlated between confocal and electron microscopy (arrowheads). The dashed square represents the area imaged in the panel below. Scale bar: 5 µm. **b** Lower panel: TEM micrographs of the rosette outlined in the upper panel (box, yellow arrow head). The boxed region is displayed at a higher magnification in the bottom images. The area containing ribosomes is highlighted in blue. Single or small groups of ribosomes are represented by individual blue dots. Outline of the rosettes is marked in yellow. The images on the right are a 3D reconstruction of the rosette with ribosomes located at the periphery of the full rosette. The 3D model is 1.54 µm thick and composed of 22 sections. Scale bars: first line 1 µm, second line 100 nm; 3D reconstruction: 1 µm. **c** Confocal images of a smiFISH experiment using a specific probe set against β-actin mRNA (green) in NIH-3T3 WT and NIH-3T3-Src cells. β-Actin protein was immnunolabeled in red. Panels on the right show enlarged views of the boxed regions. Scale bars: 10 µm. **d** NIH-3T3-Src cells were treated or not with 40 nM anisomycin for 30 min. Cells were then stained for puromycin (green) to label translating ribosomes and F-actin in red. Panels on the right show enlarged views of the boxed regions. Scale bars: 10 µm

To investigate the presence of an intrinsic and specific translational activity within invadosome rosettes, we first analyzed the endoplasmic reticulum (ER) organization associated with the rosettes. Interestingly, we noticed a concentration of ER in the center of the rosette (Fig. 4a). An orthogonal view demonstrated that the ER forms an extension, which reaches the center of the rosette from the top. Moreover, we observed a fine staining that surrounds the inner and outer parts of F-actin (Fig. 4a). A similar pattern was observed for several proteins enriched in invadosomes such as eEF2 (Supplementary Fig. 4b) and the initiation factor 4E (eIF4E), a major translation initiation factor (Supplementary Fig. 7a). We then performed correlative light and electron microscopy (CLEM) studies on NIH-3T3-Src with labeled invadosomes and observed an enrichment of ribosomes in the periphery of invadosome rosettes (Fig. 4b), demonstrating that these structures contain the required molecular machineries for performing efficient translation. In parallel, using oligo-dT probes and oligo-dA as control, we confirmed the accumulation of mRNA in invadosome rosettes (Supplementary Fig. 7b). As actin is the main structural component of these structures, we decided to test the presence of actin mRNA in invadosomes. Using single-molecule inexpensive fluorescence in situ hybridization (smiFISH)^35^, we found specific accumulation of actin mRNA in invadosome rosettes in NIH-3T3-Src cells (Fig. 4c).

In some structures such as P-bodies, despite mRNA accumulation, their translation is inhibited^36^. Therefore, we used the ribopuromycylation method (RPM) to directly visualize translation in live cells. RPM is based on incorporation of puromycin (PMY) into nascent polypeptide chains, whose association with ribosomes is maintained by the presence of the chain elongation inhibitor emetine^35^. We found a strong PMY signal around the nuclei but also in invadosome rosettes demonstrating that active protein translation took place in rosettes (Fig. 4d). This signal was abolished using anisomycin, a translation inhibitor. Consequently, we demonstrated that an internal protein translational activity is an inherent property of these invasive structures that is important for their maintenance. Overall, these data suggest that invadosomes require localized synthesis of actin to maintain their structure.

## Discussion

So far, there have been very few studies that defined the invadosome proteome, since such analyses are hindered by the structural dynamics and cumbersome isolation of invadosomes. All the previous studies used an MS-based discovery approach. Cervero et al.^10^ used a protocol for adhesive fraction from primary human macrophages allowing enrichment of the ventral membrane of the cells containing podosomes. Following relative quantification, they compared preparations of ventral membranes from macrophages bearing podosomes versus ventral membranes from PP2-treated macrophages that did not form podosomes. They identified 203 proteins (with ≥ 1 peptide) comprising 33 established podosome proteins and highlighted WDR1/AIP-1 and hnRNP-K as new components that localized to the core structure of macrophage podosomes^10^. In another study, Attanasio et al.^13^ compared invadopodia-enriched subcellular fractions with either cytosolic fractions or whole cell lysates by a difference gel electrophoresis–Matrix Assisted Laser Desorption Ionisation (MALDI) approach. Among the 58 identified proteins, they revealed new invadopodia components (14-3-3ɛ, G protein β1 subunit, GAPDH, G6PD, LDHA, and PKM)^13^, which, however, were not validated in further studies. Although these studies did not use the same invadosome models and methodologies, we have identified 12 proteins (21%) in common with Attanasio et al.^13^ and 71 proteins (35%) with Cervero et al.^10^ (Supplementary Fig. 8). Our method not only enabled the identification of more proteins than in the other two studies (570 proteins identified with ≥ 1 peptide, 366 proteins identified with ≥ 2 peptides), the selectivity of the laser microdissection approach also allowed confident interpretation of the results. Although Cervero et al.^10^ also identified several ribonuclear components and RNA-binding proteins but expressed reservation that these could be due to a defect in their preparation, we were able to demonstrate that translation-related proteins are functionally relevant invadosome components

It will be interesting to test our methodology on other types of invadosome organization, such as invadopodia (dots) observed in tumor cells or in linear invadosomes that are induced by type I collagen fibrils. For this, we will have to adapt our strategy by reducing the microdissected areas. Indeed, the laser microdissection resolution is compatible with the dimension of invadosome dots with a diameter of 1 µm for the actin core. Moreover, slices of the cell will be needed to eliminate the dorsal part, and to reduce the contamination of cytoplasmic proteins.

In this study, we combined laser microdissection and MS to enable subcellular proteomics analyses. The only comparable approach is the proteome study of Lewy bodies that are similar in size to one or several cells^37^. Laser microdissection, facilitated by the automation strategy presented here, enables the highly specific selection of a cellular compartment of interest. With such a controlled technical process, we can now analyze the proteome of very small compartments (even sub-organellar compartments). It also raises possibilities for the analysis of other technically challenging membrane structures, such as cellular junctions. Moreover, our method offers the advantage to be suitable for fixed samples, allowing the possibility to analyze a specific maturation step correlated to a specific morphology as demonstrated with invadosome rosettes.

We believe that combining laser microdissection and MS has the potential to solve subcellular proteomics challenges by guaranteeing specificity and sensitivity. We provide an alternative to the classical biochemical fractionations, known for their limitations both in terms of contaminations by other organelles and the impossibility to robustly analyze membranes structures or other elements destabilized or destroyed by cell lysis. As our method allows analyzing and deciphering the proteome of targeted subcellular compartments in their native state, it may also provide alternative solutions for the analysis of protein complexes classically investigated by immunoprecipitation.

Our approach allowed us to demonstrate an internal protein translational activity associated with invadosomes. We showed that global inhibition of the translational machinery inhibits invadosome formation. Moreover, we localized several proteins identified by proteomics to invadosomes. Their depletion using an siRNA screen blocked invadosome formation and/or activity. To confirm the presence of a local and active translation activity into invadosome rosettes, we have shown (i) the presence of ribosomes using CLEM, (ii) an accumulation of mRNA and especially actin mRNA, and (iii) an active translation activity into invadosomes. Although some technical aspects of CLEM have to be further improved, such as the *Z*-correlation and the preservation of the invadosome ultrastructure to keep actin filaments, this analysis has proven valuable to substantiate the implications of our proteomics analysis, which associates invadosomes with a specific translation machinery. The various protein elements of this machinery could represent new targets to block matrix degradation and cancer cell invasion. Moreover, the invadosome proteome presented here can serve as a resource to better understand molecular mechanisms involved in invadosome formation and activity.

During our study, we showed that depletion of proteins implicated in the translation decreases the cell’s ability to form invadosomes and degrade the extracellular matrix (ECM). Interestingly, caprin 1 depletion impacts only the degradation activity and does not affect the number of invadosomes in two different models. This result suggests that caprin 1 impacts directly and specifically the invadosome degrading machinery and confirms that these two aspects, actin structure, and degradation activity of the invadosome, are not systematically linked^38^.

We also observed that the localization of translational-related proteins like eEF2 or eIF4E can be different, suggesting that their role could differ depending on the maturation step of the invadosome. It will be important to investigate their association with newly forming or actively degrading structures. This relocation of translation-related proteins is coherent with the dynamics of invadosome structures and was already described for other subcellular compartments in neurons^39^. Indeed, intricate regulation of compartment-specific mRNA translation in mammalian central nervous system axons supports the formation and maintenance of neural circuits in vivo. Moreover, the presence and the translation of β-actin mRNA in filopodia and lamellipodia is known for a long time^40^. Invadosome rosettes are dynamic structures formed by a constant polymerization–depolymerization activity of actin filaments, which is needed to ensure generation, maturation, stabilization, and collapse of the structure. Conceivably, this continuous reorganization requires subcellular mRNA accumulation and local protein translation. Now, it is clear that β-actin mRNAs are not the only mRNA translated into these invasive sites. The next step will be to identify the mRNAs specifically translated into invadosomes to fully understand the implication of this process in invadosome formation and more globally in cancer cell invasion.

The local concentration of proteins is a limitation to cellular processes^41^. Likewise, translational control is a crucial component of cancer development and progression. The control of protein synthesis and the selection of specific mRNAs are involved in cancer invasion and metastasis^31^. Notably, some mRNA-binding proteins identified with our methodology are already associated with cancer progression and invasion (Supplementary Table 3 and Supplementary Data 2). In the future, this invadosome feature, which remains to be tested in vivo, could pave the way for the identification of a translational signature for tumor cell invasion, which could then be pharmacologically targeted.

## Methods

### Cell culture

NIH-3T3 WT and NIH-3T3-Src cells were a generous gift from Sara A. Courtneidge (Burnham Institute for Medical Research, LaJolla, CA). The cells were maintained in Dulbecco’s modified Eagle’s medium (DMEM) with 4.5 g/L glucose Glutamax-I (Invitrogen) supplemented with 10% fetal calf serum (Sigma-Aldrich) and 100 U/mL penicillin–streptomycin (Invitrogen). Huh6 cells (human hepatoblastoma cell line) were provided by C. Perret (Cochin Institute, Paris, France) and cultured in DMEM medium with 1 g/L glucose Glutamax-I (Invitrogen) supplemented with 10% fetal calf serum (Sigma-Aldrich) and 100 U/mL penicillin–streptomycin (Invitrogen). Huh6 cell-line authentication was done by STR Matching analysis in comparison with the JCRB0401 cell line (evaluation value = 0.97 (32/33)). All the cell lines were confirmed for the absence of mycoplasma by PCR.

### Transfection

Caprin-myc, eIF3L-myc, HA-eEF1A1, and eEF2-GFP were purchased form Origene. Those plasmids were transfected (1 µg) using JetPrime (PolyPlus Transfection) following the manufacturer’s instructions. siRNA oligonucleotides (60 nM) were transfected using the Lipofectamine RNAiMax (Invitrogen) according to the manufacturer’s instructions. The sequences are listed in the Supplementary Table 4 for NIH-3T3-Src. For assays with Huh6 cells, the sequences are Hs_sieEF2#1: 5′-CCGCGCCATCATGGACAAGAA-3′ and Hs_sieEF1A1#: 5′-AAGGAATATCATTTAAAGCTA-3′.

### Antibodies and reagents

The anisomycin and the CHX were purchased from Sigma-Aldrich. The GM6001 was purchased from EMD Millipore. PP2, eEF1A1 antibody (EPR9470, catalog number ab140632), and anti-GFP (LGB-1, catalog number ab291) were purchased from Abcam. Anti-Myc (9E10, catalog number sc-40) was purchased from Santa Cruz Biotechnology, Inc. Anti-eIF3H (D9C1, catalog number 3413) was purchased from Cell Signaling Technology. Anti-HA (3F10, catalog number 12158167001) was purchase from Sigma-Aldrich. All primary antibodies have been diluted at 1:100 for immunofluorescence. Secondary antibodies FluoProbes 488 anti-rabbit and anti-mouse (FP-SA4110, FP-SA5110), and 547H anti-rabbit and anti-mouse (FP-SB4110, FPSB5110) were purchased from Interchim, and diluted at 1:200 for immunofluorescence. Hoechst 34580 (Invitrogen) was used to stain nuclei. For the ER localization, cells were incubated with 1 µM of ER-Tracker^TM^ green (BOPIDY^TM^ FL Glibenclamide) (Molecular Pobes^TM^) for 30 min at 37 °C. Then the cells were fixed in 4% PFA before confocal imaging.

### In situ zymography assay

Coverslips were coated with Oregon green gelatin (Invitrogen), fixed with 0.5% glutaraldehyde (GA) (Electron Microscopy Sciences), and washed with phosphate-buffered saline (PBS) (Invitrogen). Cells were seeded on coated coverslips and incubated 24 h before fixation and staining.

### Immunofluorescence

Cells were fixed with 4% PFA, pH 7.2, for 10 min, permeabilized with 0.2% Triton X-100 for 10 min, and incubated with various antibodies, as described earlier in Juin et al^4^. Cells were imaged with a SP5 confocal microscope (Leica, Leica Microsystems GmbH, Wetzlar, Germany) by using a × 63/numerical aperture (NA) 1.4 Plan Neofluor objective. To prevent contamination between fluorochromes, each channel was imaged sequentially using the multitrack recording module before merging.

### Statistical analysis

Statistical analyses were performed using GraphPad Prism 6.0 software. Regular one‐way analysis of variance (ANOVA) was used for the comparison of multiple means. Means were considered significantly different when *P* < 0.05. The ANOVA test was followed by a Bonferroni’s multiple‐comparison post test, each condition was compared with the control.

### SILAC labeling

NIH-3T3-Src cells were grown in DMEM medium without lysine and arginine (Gibco) supplemented with 10% dialyzed fetal bovine serum (Gibco), 200 mg/L l-proline (Sigma-Aldrich), and 84 mg/L ^13^C6 l-Arginine (R) and 146 mg/L ^13^C6 l-Lysine (K) (both from Eurisotop) at 37 °C in a humidified incubator in a 5% CO_2_ atmosphere. The total incorporation of the labeling was checked by MS after six cycles of cellular doubling.

### Laser microdissection

Invadosomes were microdissected from PFA fixed Src-NIH-3T3 cells with a PALM type 4 (Zeiss) automated laser microdissector. Five analyses were performed with an increasing number of microdissected invadosomes (100, 350, 3000, 10,000, 40,000). The four first ones were manually made. The microdissection of 40,000 rosettes was made using the system of automation that we developed.

### Assisted invadosomes laser microdissection

An ImageJ macro toolset was used to automatically segment invadosomes and export the ROI to be isolated toward the PALM Zeiss microdissector. Although the sample is being scanned, the ImageJ toolset monitors the arrival of newly saved images. For each single image, pre-processing and segmentation steps are performed as follows. First, the channels from the red-green-blue (RGB) images are split: only the red image is retained. A median filtering is applied (radius: 2 pixels) and a background subtraction is performed using the rolling-ball algorithm (radius: 20 pixels). To isolate invadosomes from background, automated thresholding is applied, morphological closing and hole filling performed. Finally, structures are delineated by connexity analysis (ImageJ analyse particles function). Morphological parameters, strictly defined on well-identified structures, are used to only retain structures of interest: objects’ area should be at least 10 µm^2^, with a circularity enclosed within the 0.35–1 range. This analysis output takes the form of ROI, stored within the ImageJ RoiManager. All ROIs are exported as an “Element file”, using an in-house developed ImageJ plugin.

### High content analysis

Images for high content analysis were collected using an inverted Leica DMI 6000 microscope (Leica Microsystems) equipped with a HC PLAN APO × 20/0.7 objective, a Lumencor spectra 7 illumination device (Lumencor, Beaverton, USA) and a HQ2 charge-coupled device (CCD) camera (Photometrics, Tucson, USA). This system was under the control of the MetaMorph software (Molecular Devices, Sunnyvale, USA), under which a series of journal has been created to automate the acquisitions over a multi-well plate (24 wells/plate, 25 fields imaged/well). Automated image analysis has been performed using an in-house developed workflow, using the CellProfiler software^42^. Briefly, for both the invadosomes and nuclei images a correction for uneven illumination has been performed. Invadosomes were first enhanced by top-hat filtering. Candidate structures were isolated using the “Identify primary objects” function, then submitted to a refined identification by filtering based on morphological parameters (area, major/minor axis length, and solidity). Nuclei identification was performed using only the primary object detection. For both channels, an image of the objects outlines was saved to visual assess the segmentation efficiency. All morphological parameters were extracted and saved as a SQLite database for further reviewing and analysis using the Cell Profiler Analyst software^43^.

### Range of protein quantity of total cellular extract

SILAC labeled NIH-3T3-Src cells were lysed in RIPA buffer (Sigma) supplemented with protease inhibitor cocktail (Roche). Protein concentrations were measured using the Bio-Rad protein assay.

### Sample preparation for MS

Micro-dissected invadosomes and whole cells extracts were incubated in a Tris-HCl pH 6.8 solution for 2 h at 95 °C. Samples were loaded on a 10% acrylamide SDS-polyacrylamide gel electropjoresis (PAGE) gel. Migration was stopped when the samples entered the resolving gel and the proteins were visualized by colloidal blue staining. The SDS-PAGE band was cut into 1 mm × 1 mm gel pieces. Gel pieces were destained in 25 mM ammonium bicarbonate (NH_4_HCO_3_), 50% acetonitrile (ACN) and shrunk in ACN for 10 min. After ACN removal, the gel pieces were dried at room temperature. The proteins were first reduced in 10 mM dithiothreitol, 100 mM NH_4_HCO_3_ for 30 min at 56 °C then alkylated in 100 mM iodoacetamide, 100 mM NH_4_HCO_3_ for 30 min at room temperature, and shrunk in ACN for 10 min. After ACN removal, the gel pieces were rehydrated with 100 mM NH_4_HCO_3_ for 10 min at room temperature. Before protein digestion, the gel pieces were shrunk in ACN for 10 min and dried at room temperature. The proteins were digested by incubating each gel slice with 10 ng/µL of trypsin (T6567, Sigma-Aldrich) in 40 mM NH_4_HCO_3_, 10% ACN, rehydrated at 4 °C for 10 min, and were finally incubated overnight at 37 °C. The resulting peptides were extracted from the gel in three steps: the first incubation was in 40 mM NH_4_HCO_3_, 10% ACN for 15 min at room temperature, and two subsequent incubations were in 47.5% ACN, 5% formic acid for 15 min at room temperature. The three collected extractions were pooled with the initial digestion supernatant, dried in a SpeedVac, and re-suspended with 20 μL of 0.1% formic acid before nanoLC-MS/MS analysis.

### MS analysis

Online nanoLC-MS/MS analyses were performed using an Ultimate 3000 RSLC Nano-UPHLC system (Thermo Scientific, USA) coupled to a nanospray Q-Exactive hybrid quadrupole-Orbitrap mass spectrometer (Thermo Scientific). Ten microliters of the peptide extract were loaded on a 300 µm ID × 5 mm PepMap C18 precolumn (Thermo Scientific) at a flow rate of 20 µL/min. After 5 min desalting, peptides were separated on a 75 µm ID × 25 cm C18 Acclaim PepMap® RSLC column (Thermo Scientific) with a 4–40% linear gradient of solvent B (0.1% formic acid in 80% ACN) in 108 min. The separation flow rate was set at 300 nL/min. The mass spectrometer operated in positive ion mode at a 1.8 kV needle voltage. Data were acquired using Xcalibur 3.1 software in a data-dependent mode. MS scans (m/z 350–1600) were recorded at the resolution of *R* = 70,000 (@ m/z 200) and an AGC target of 3 × 106 ions collected within 100 ms. Dynamic exclusion was set to 30 s and top 12 ions were selected from fragmentation in HCD mode. MS/MS scans with a target value of 1 × 105 ions were collected with a maximum fill time of 100 ms and a resolution of *R* = 17,500. In addition, only + 2 and + 3 charged ions were selected for fragmentation. The other settings were as follows: no sheath and no auxiliary gas flow, heated capillary temperature 200 °C, normalized HCD collision energy of 27%, and an isolation width of 2 m/z.

### MS data processing and quantification

For protein identification, we used the Mascot 2.5 algorithm available with Proteome Discoverer 1.4 Software (Thermo Fisher Scientific, Inc.). It was used in batch mode by searching against the UniProt *Mus musculus* database (45,172 entries, Reference Proteome Set, release 2016_03) from [http://www.uniprot.org/website]. Two missed enzyme cleavages were allowed. Mass tolerances in MS and MS/MS were set to 10 p.p.m. and 0.02 Da. Oxidation of methionine, acetylation of lysine, and deamidation of asparagine and glutamine were looked for dynamic modifications. Carbamidomethylation on cysteine was searched as static modification. ^13^C(6) (K) or ^13^C(6) (R) labeling was searched as variable modification for contaminating analyses and as fixed modification for invadosomal proteome analyses.

For the enrichment analysis compared with the total cellular proteome, raw LC-MS/MS data were imported in Proline Studio (http://proline.profiproteomics.fr/) for feature detection, alignment, and quantification. Protein identification was only accepted with at least 2 specific peptides with a pretty rank = 1 and with a protein false discovery rate value < 1.0% calculated using the “decoy” option in Mascot^44^. Label-free quantification of MS1 level by extracted ion chromatograms was carried out using the parameters indicated in Supplementary Table 5. Protein ratios were normalized by sum of peak intensities. Proteins with invadosomes/total ratios ≥ 1.5 were considered as enriched in comparison with the whole cellular proteome.

### Bioinformatics analysis

Proteins listed in the Supplementary Table 2 and Supplementary Data 1 were used for analyses of GO (Protein class) classification from the PANTHER (http://pantherdb.org) database.

### smiFISH analysis

smiFISH was performed as described^35^. Briefly, cells were fixed with 4% PFA and permeabilized in 70% ethanol. Cells were then hybridized using two types of probes: (i) 24 unlabelled primary probes containing both a β-actin mRNA targeting sequence and a shared sequence (FLAP); (ii) a secondary probe conjugated to two Cy3 moieties, pre-hybridized in vitro to the primary probes via the FLAP sequence. All probes were purchased from Integrated DNA Technologies. The probe sequences are listed in Supplementary Table 6. Following smiFISH, cells were permeabilized with 0.1% Triton X-100 and stained with anti-β-actin (clone AC-15, Sigma A5441) and goat anti-mouse-FITC (Jackson ImmunoResearch 115-095-062) antibodies^35^.

### Microscopy

Three-dimensional (3D) image stacks were captured on a wide-field microscope (Zeiss Axioimager Z1) equipped with a × 63/1.4 NA objective and a sCMOS camera (Zyla 4.2 MP, Andor Technology) and controlled with Metamorph (Molecular Devices). Maximum intensity projections of image stacks were obtained with ImageJ (Rasband, W.S., ImageJ, U. S. National Institutes of Health, Bethesda, Maryland, USA, [https://imagej.nih.gov/ij/, 1997-2016]). For confocal microscopy, cells were imaged with a SP5 confocal microscope (Leica, Leica microsystems GmbH) using a × 63/NA 1.4 Plan Neofluar objective lens. Images correspond to optical slice with a pinhole of 95.54 µm. To prevent fluorochromes crosstalk contamination, each channel was imaged sequentially using the multitrack recording module before merging. *Z*-stack pictures were obtained using LAS AF, Leica software. The 3D reconstructions were obtained from Z-slices pictures, by using Leica software (LAS AF, Leica Software, Germany)^4^. Correlative microscopy, for performing CLEM of rosettes, we used laser micro-patterned aclar films^45^. The aclar films were pre-coated with 0.5 mg/mL of gelatin in a 24-well plate. NIH-3T3 cells were seeded at 20,000 cells/mL and incubated overnight. The next day, the aclar film was transferred to a 35 mm glass bottom dish (MatTek P35G-1.0-20-C) for confocal imaging (Leica SP2) using a × 63 objective (NA:1.32, Leica Microsystems). High-magnification fluorescent pictures of cells and rosettes were acquired. In addition, low-magnification images were acquired for mapping the position of the cell in relation to the micro-patterns that are visible using transmitted light (see experiment workflow). Once imaged, cells were fixed in 2.5% PFA (EMS 15713) and 2.5% GA (EMS 16220) in 0.1 M cacodylate buffer (EMS 11652) for 1 h at room temperature. The cells were post-fixed in 1% OSO_4_ (EMS 19150) in cacodylate buffer 0.1 M for 1 h on ice. After 3 × 10 min water rinses, cells were stained with 2% uranyl acetate. The cells were dehydrated in sequential gradient alcohol baths and infiltrated with epon (resin). Plastic embedding capsule (EMS 69910-05) were mounted over the aclar film following overnight polymerization at 60 °C. The following day, the capsules were filled with epon and polymerized again overnight at 60 °C.

For relocating the region of interest, the surface of the resin block was imaged using a stereomicroscope allowing accurate positioning of the cell of interest according to the micro-pattern. Precise trimming was performed around the cell of interest. Finally, the resin block was serially sectioned (thickness: 70 nm) and all the sections were collected on electron microscope slot grids. Sample sections were imaged on a CM12 transmitted electron microscope (FEI company) with a CCD camera (Orius, Gatan). For the image processing, fluorescent images were processed in Fiji^46^ and electron microscopy images were aligned and stacked with TrakEM2^47^. The 3D rendering of the electron microscopy acquisitions were performed with Imod^48^.

### RPM method

To visualize newly synthetized proteins within cells, we used the RPM method as described by David et al^49^. NIH-3T3-Src cells grown on coverslips were incubated for 15 min at 37 °C in complete H12 medium supplemented with 208 µM emetin (EMD, Sigma). In protein synthesis inhibitor control experiments, cells were pretreated with 40 µM anisomycin (Sigma) for 30 min at 37 °C before incubation with emitin. Cells were then treated with 355 µM CHX (Sigma) for 2 min on ice in permeabilization buffer (50 mM Tris-HCl, pH 7.5, 5 mM MgCl_2_, 25 mM KCl, 0.015% digitonin, EDTA-free protease inhibitor, 10 U/mL RNaseOut (Invitrogen)). Cells were then washed and incubated in polysome buffer (50 mM Tris-HCl, pH 7.5, 5 mM MgCl_2_, 25 mM KCl, 0.2 M sucrose, EDTA-free protease inhibitor, 10 U/mL RNaseOut (Invitrogen)) supplemented with 91 µM PMY (Sigma) for 10 min on ice. After rapid washing in polysome buffer, cells were fixed in 4% PFA for 10 min at room temperature. After fixation, cells were washed twice with PBS and immunostaining with anti-PMY antibody was performed as described above.

### Code availability

The in-house developed ImageJ plugin is available from https://github.com/fabricecordelieres/IJ_PALM_Zeiss_workflow.

### Data availability

The MS proteomics data were deposited in the ProteomeXchange Consortium via the PRIDE partner repository with the dataset identifier PXD009390 [https://www.ebi.ac.uk/pride/archive/projects/ PXD009390]. All other data supporting the findings of this study are available from the corresponding author on reasonable request.

## Electronic supplementary material

Supplementary Information

Peer Review File

Description of Additional Supplementary Files

Supplementary Movie 1

Supplementary Data 1

Supplementary Data 2
