# Supplementary material for: Combining laser capture microdissection and proteomics reveals an active translation machinery controlling invadosome formation
Source: Nat Commun. 2018 May 23;9:2031. doi: 10.1038/s41467-018-04461-9 (PMC5966458; doi:10.1038/s41467-018-04461-9)
Supplement: Supplementary file 1 — Supplementary Information [file 41467_2018_4461_MOESM1_ESM.pdf]

Combining laser capture micro-dissection and proteomics reveals an active translation machinery controlling invadosome formation

Ezzoukhry and Henriet et al.

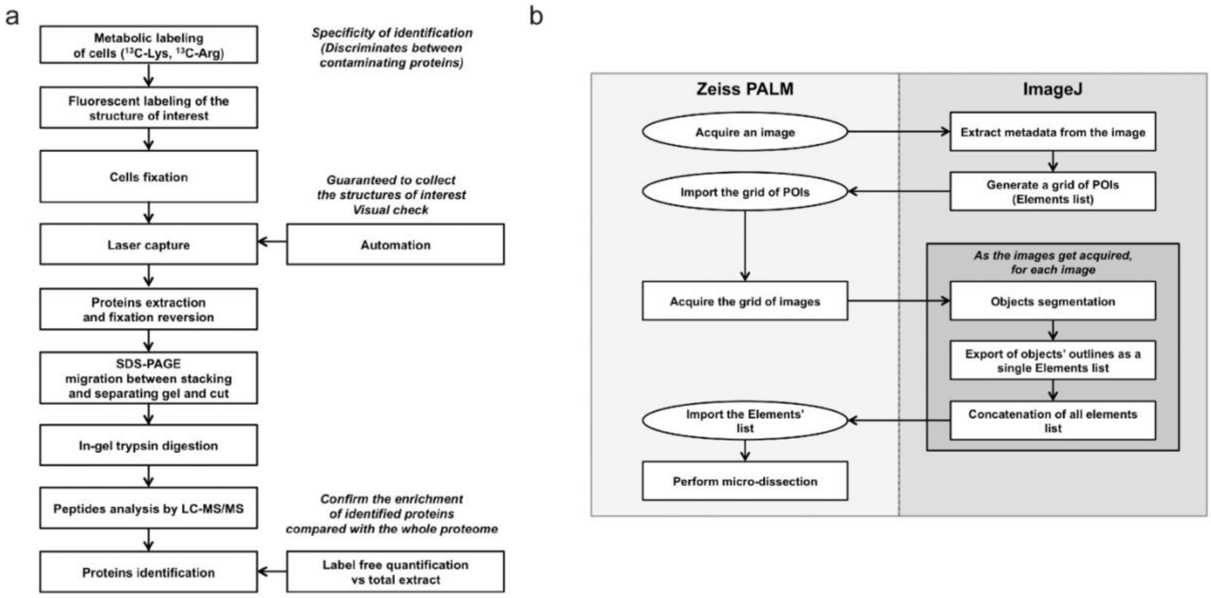

Supplementary Fig. 1: Flowchart of the analytical process
(a) Technical flow chart including the metabolic labeling of the proteins to ensure specificity of identification, isolation and collection of fluorescent-labeled structures of interest with an automated laser capture, and finally, protein identification by LC-MS/MS analysis and the enrichment quantification by a label free approach. (b) Detailed workflow of the automated laser capture. A first image is acquired from the Zeiss PALM software. Its positional information is extracted and used by an ImageJ plugin in order to generate a grid of coordinates, covering adjacent field to be explored. For each field, an image is acquired and automatically analyzed by ImageJ to recover the outlines of structures of interest. Structure contours are exported from the ImageJ software as a Zeiss PALM compatible file. The micro-dissection step is started upon this file's import. Automatic steps are indicated in squares and manual steps in circles.
(b) Assisted invadosome micro-dissection workflow.

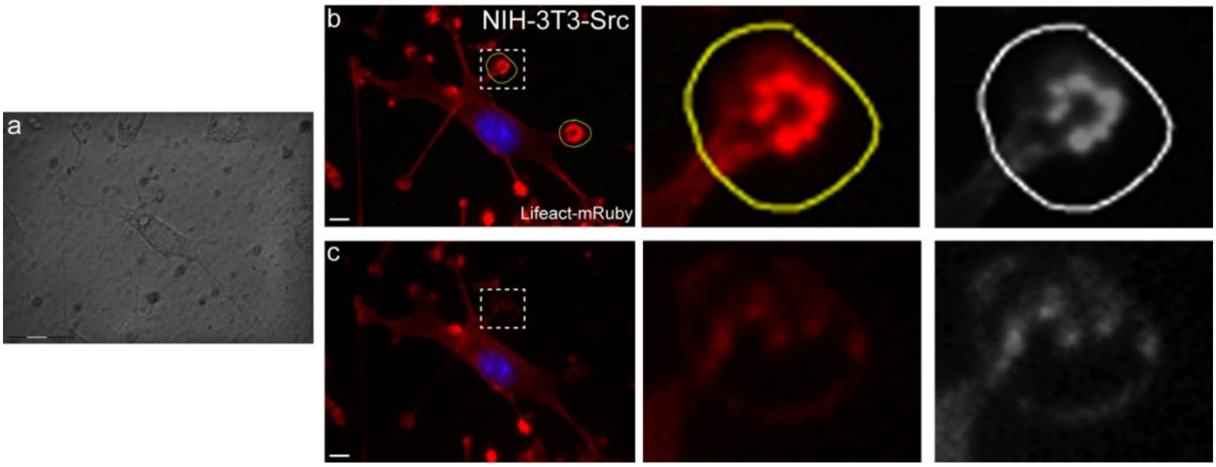

Supplementary Fig. 2: Invadosome laser microdissection
Representative PALM Zeiss microdissector images of lifeact-mRuby (red)-expressing NIH-3T3-Src cells by transmitted light (a) (scale bar: 30µm). In the higher image (b), the dotted circles surround the rosettes that will be micro-dissected. Blue is Hoechst nuclear staining. The lower image (c) shows the cell after microdissection. Right panels show enlarged views of the boxed regions (scale bar: 10µm).

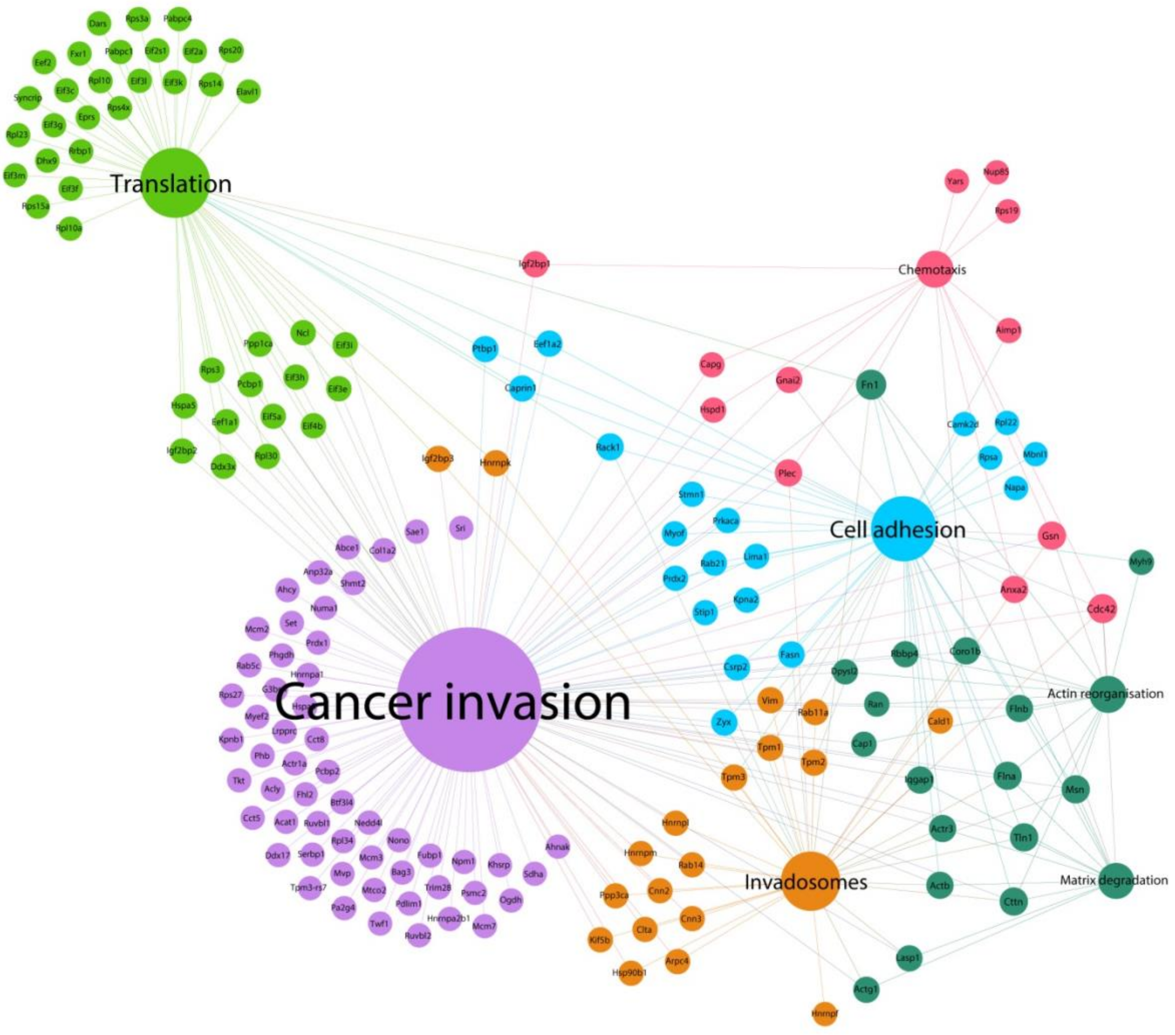

Supplementary Fig. 3: Functional network of proteins identified in invadosomes and enriched compared to the whole cellular proteome. Annotations were attributed manually for the following functions: “Cancer invasion”, “Invadosomes”, and “Matrix degradation”. Involvement in actin reorganization, cell adhesion, chemotaxis or protein translation was extracted from the Ingenuity® Pathway Analysis Database (Qiagen). Networking was made with the Gephi software. Individual proteins (small circles) are grouped according to depicted molecular functions (large circles) and color-coded. Proteins that can be attributed to several groups are linked between groups.

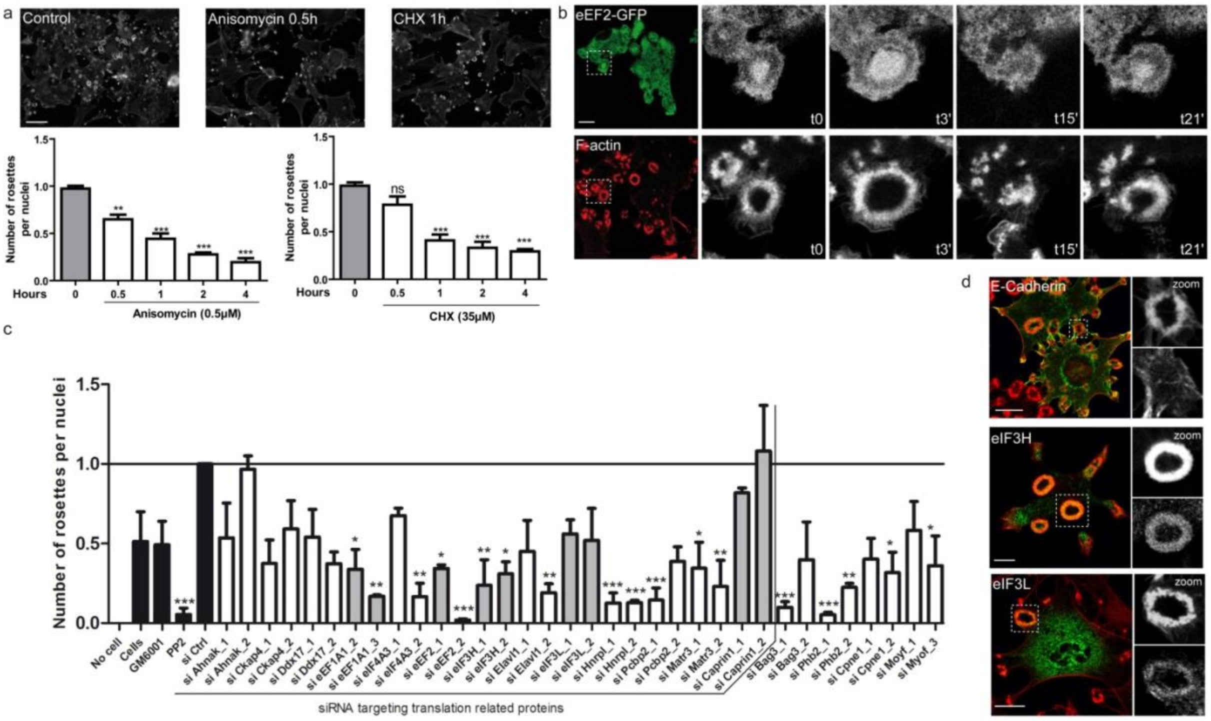

Supplementary Fig. 4: Localization and involvement of translation-related proteins

(a) Time-course of the number of rosette per nuclei after translation inhibitor treatment. Lifeact-mRuby-expressing NIH-3T3-Src cells were treated with 0.5 µM anisomycin or 35 µM CHX for the indicated time points. Panel on the top shows representative images of the cells for which there is an effect at the earlier time point. The bar graph represents the number of rosettes per nuclei. Error bars represent the SEM (n=20 fields, three independent experiments; ns, not significant, **, P < 0.005; ***, P < 0.001 as compared to the non-treated cells as control). Scale bar: 50 µm.

(b) Representative images from time-lapse video microscopy of lifeact-mRuby (red)-expressing NIH-3T3-Src cells transfected with eEF2-GFP (green). Scale bar: 10 µm.

(c) siRNA screening targeting 19 of the most enriched candidate proteins. Bar graph shows the number of rosettes per nuclei. The black bars represent the controls of the experiment. Control cells were treated with 5 µM GM6001 (metalloproteinase inhibitor) or 5 µM PP2 (Src inhibitor) or with a control siRNA (siCtrl). The grey bars represent the proteins that were further localized. Error bars represent the SEM (n=75 fields, three independent experiments; ns, not significant; *, P <0.05; **, P < 0.005; ***, P < 0.001 by One-way ANOVA followed by Bonferroni test as compared to the siRNA control).

(d) Confocal images of lifeact-mRuby (red)-expressing NIH-3T3-Src cells transfected with E-Cadherin-GFP or eIF3L-myc (green) and eIF3H (green) revealed by indirect immunofluorescence. Panels on the right show enlarged views of the boxed regions in black and white for each channel (Lifeact-mRuby at the top and the protein of interest at the bottom). Scale bars: 10 µm.

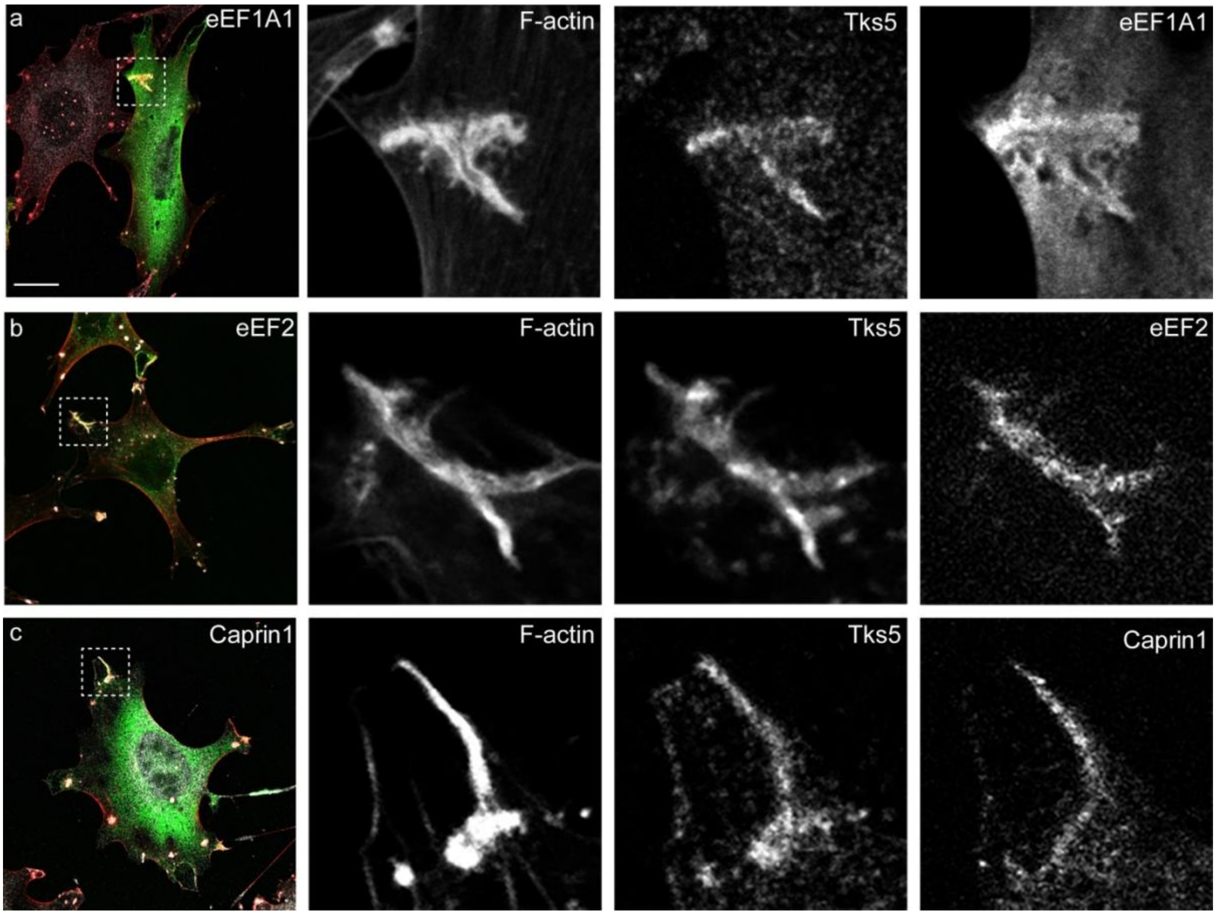

Supplementary Fig. 5: Translation-related proteins at invadosomes in linear invadosome
Representative images of Lifeact-mRuby (red)-expressing NIH-3T3-Src seeded on a fibrillar type I collagen matrix and presented linear invadosomes. Those cells were transfected with (a) HA-eEF1A1, (b) eEF2-GFP or (c) Caprin1-myc and processed for fluorescent staining: HA, GFP or Myc (green) and Tks5 (grey). The 3 images on the right show enlarged views of the boxed regions in black and white for each channel. Scale bar: 5 µm.

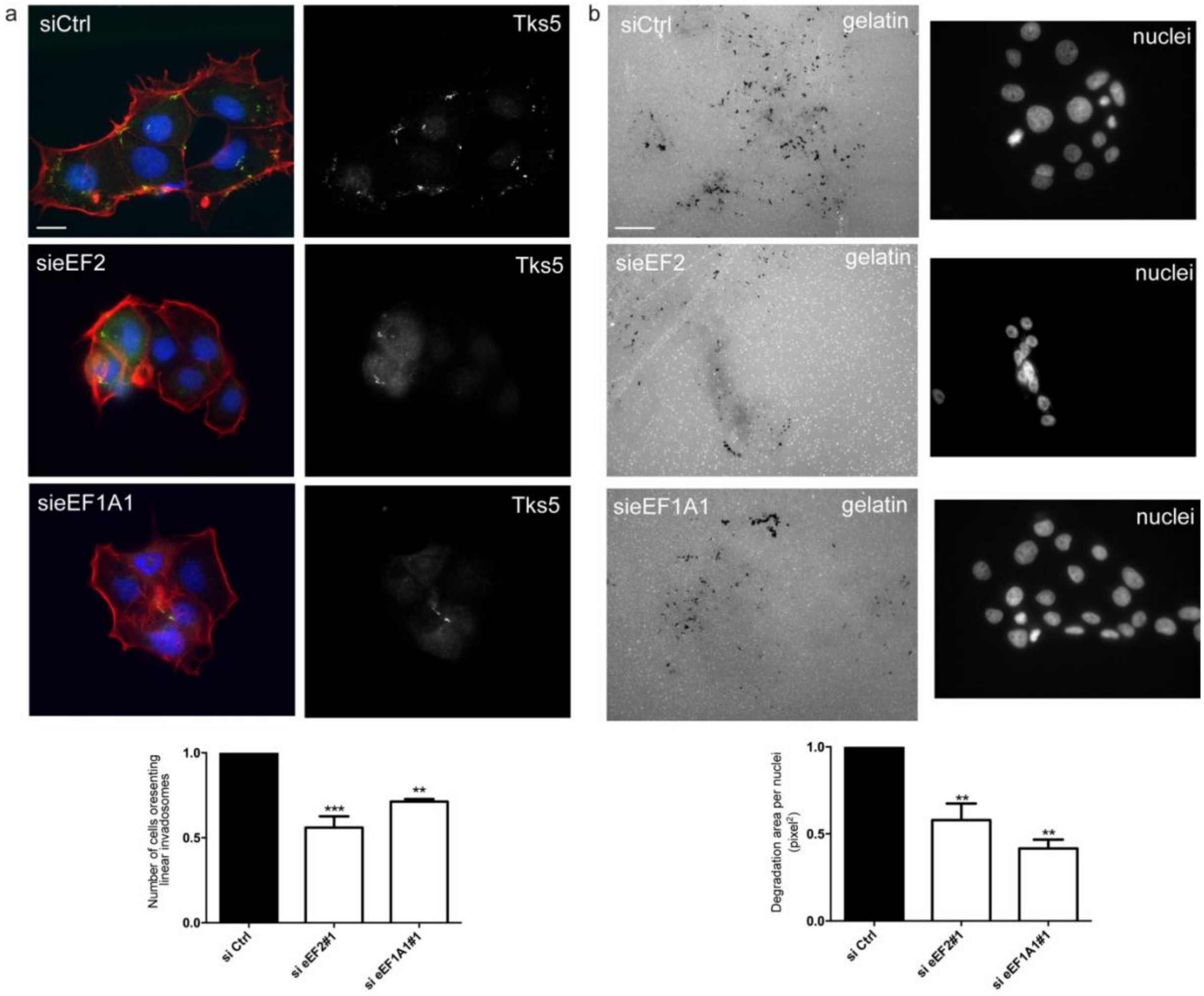

Supplementary Fig. 6: Involvement of translation-related proteins at invadosomes in Huh6
(a) Huh6 cells were transfected with a siRNA control (siCtrl) or siRNA targeting eEF2 or eEF1A1 involved in translation activity. The cells were then seeded on a fibrillar type I collagen matrix. The top panel shows representative images of Huh6 cells presenting linear invadosomes. Scale bar: 10 µm. Bar graph shows the number of cells presenting linear invadosomes after 5 h. The black bar represents the siRNA control. Error bars represent the SEM of three independent experiments (**, $P < 0.005$; ***, $P < 0.001$ as compared to the siRNA control).
(b) Huh6 cells transfected with a siRNA control (siCtrl) or siRNA targeting eEF2, or eEF1A1 were seeded on a mix matrix of fibrillar type I collagen and fluorescent gelatin. The top panel shows representative images of the degraded area (black) and the number of nuclei in the same field. Scale bar: 10 µm. Bar graph shows the gelatin area degraded per cell after 24 h. The black bar represents the siRNA control. Error bars represent the SEM (n=30 fields, three independent experiments; **, $P < 0.005$ as compared to the control siRNA).

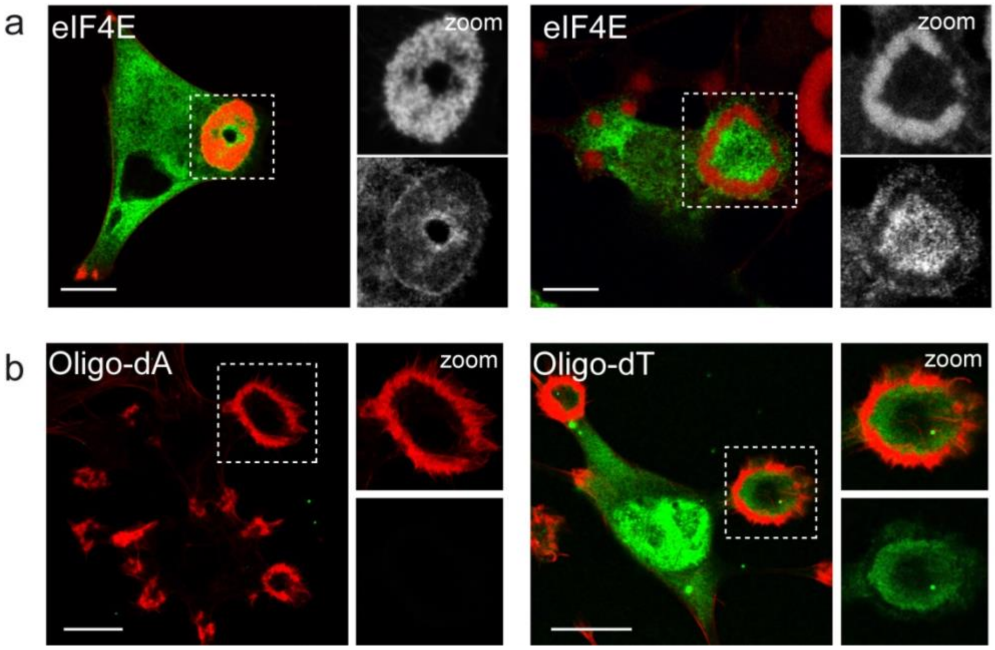

Supplementary Fig. 7: eIF4E and mRNA localize at invadosome
(a) Lifeact-mRuby (re)-expressing NIH-3T3-Src cells were transfected with a construct encoding the translation initiation factor HA-eIF4E and stained using HA antibodies (green). Panels on the right show enlarged views of the boxed regions in black and white for each channel (Lifeact-mRuby at the top and eIF4E at the bottom). (b) Lifeact-mRuby (red)-expressing NIH-3T3-Src cells were transfected with oligo-dT probes (green) revealing the presence of poly(A)-RNA in rosettes. An oligo-dA probe was used as a negative control. Panels on the right show enlarged views of the boxed regions. Scale bars: 10 µm.

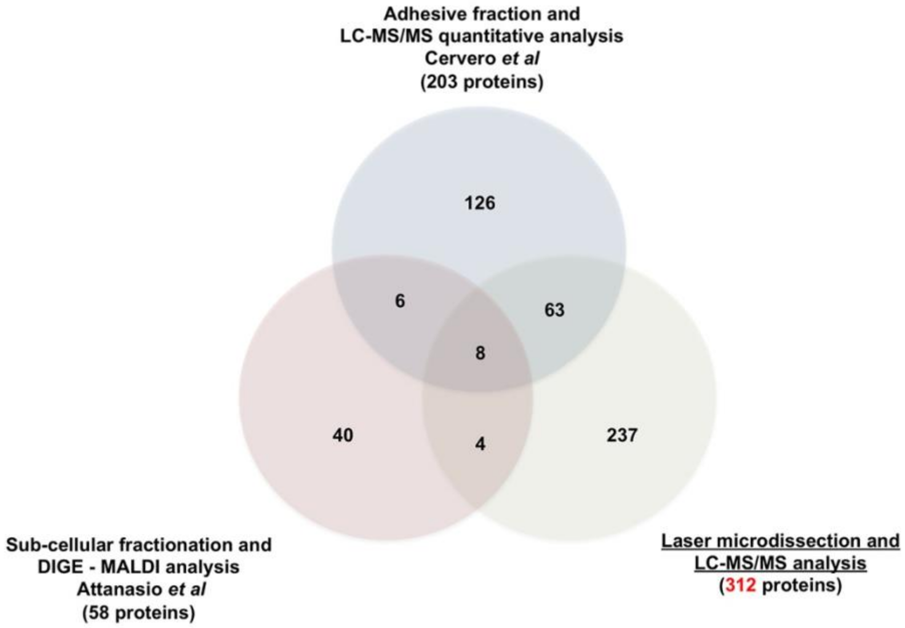

Supplementary Fig. 8: Invadosome's proteomic datasets comparison
Comparison of proteins identified in two other proteomic studies with proteins identified using our new method that combines laser micro-dissection and mass spectrometry analysis [1] [2].

Sample	Protein quantity (ng)	Number of identified peptides	Number of identified proteins ≥1 peptides	Number of identified proteins ≥2 peptides	Total surface area of ^{13}C peptides
Total proteome	1000	12555	1941	1459	2,45E+12
Total proteome	500	8015	1485	1092	7,64E+11
Total proteome	100	4334	839	627	1,54E+11
Rosettes	72	2286	570	366	4,53E+10
Total proteome	50	611	168	93	8,84E+09
Total proteome	10	37	11	6	6,46E+08

Supplementary Table 1: Global mass spectrometry results of rosettes sample analysis compared to the total proteome.
The table recapitulates the number of identified peptides, the corresponding number of identified proteins with at least 1 or 2 specific peptides and the associated sum of MS intensities of all ^{13}C peptides detected according to a range of proteins quantity from ^{13}C labelled NIH-3T3-Src cells. The sum of intensities of all detected ^{13}C peptides was used to deduce the protein quantity from the 40 000 rosettes sample.

**proteins only detected in invadosomes fraction (gene name) :**<br>Atp5d, Col1a2, Cops4, Eif3h, Eif3k, Emc2, Immt, Mcm2, Mybbp1a, Ndufs1, Numa1, Pdhb, Purb, Rab21, Rpl37a, Sdha, Snrpa, Srrt, Tomm70, Ythdf2
**invadosome/total proteome ratio ≥ 10 :**<br>Atxn2l, Bag3, Bub3, Fn1, Gm5449, Matr3, Mtcl1, Myef2, Rbm14, Rtcb, Sf3a1, Sf3b3
invadosome/total proteome ratio ≥ 5 :<br>Acat1, Aldh2, Crocc2, Eif2a, Eif3e, Elavl1, Eprs, Fubp1, Fxr1, G6pdx, Hnrnpc, Hnrnpf, Hnrnpl, Hnrnpm, Hsd17b10, Hspa4l, Igf2bp1, Lima1, Mcm3, Mthfd1l, Ncl, Nono, Psmc1, Rpl31, Rpl36a, Rps14, Ruvbl2, Sarnp, Sept9, Serbp1, Trim28
**invadosome/total proteome ratio ≥ 2 :**<br>Abce1, Acot1, Actr1a, Ahcy, Ahnak, Ahnak2, Aimp1, Anp32a, Arcn1, Atp5c1, Atp5o, Atp6v1b2, Btf3l4, Cacybp, Cald1, Camk2d, Cap1, Caprin1, Cars, Chmp4b, Ckap4, Cnn2, Cnn3, Coro1b, Cpne1, Csrp2, Cttn, Dctn1, Ddx17, Ddx3x, Ddx5, Dhx15, Dhx9, Dlst, Eftud2, Eif2s1, Eif3c, Eif3f, Eif3g, Eif3i, Eif3l, Eif3m, Eif4b, Farsa ,Fasn, Fhl2, Fkbp4 ,Flna, Flnb, Flnc, G3bp1, Gls, Gm10036, Gm28062 ,Gm6793, Gm8797, Gm9755 ,Gpd2, Grpel1, H2afj, Hadhb, Hdlbp, Hist1h2bm, Hist2h3b, Hist2h4, Hnrnpa1, Hnrnpa2b1, Hnrnph1, Hnrnpk, Hspa8, Hspa9, Hspd1, Hspe1, Hsph1, Igf2bp2, Igf2bp3, Impdh2, Iqgap1, Khsrp, Kpna2, Kpnb1, Lasp1, Lmna, Lmnb1, Lonp1, Map4, Mbnl1, Mcm6, Mcm7, Mtco2, Mthfd1, Mvp, Myh13, Myl6, Myof, Naca, Nasp, Nedd4l, Npm1, Ogdh, Ola1, Pa2g4, Pabpc1, Pabpc4, Pcbp1, Pcbp2, Phb, Phb2, Plec, Ppp3ca, Prdx2, Prpf19, Psma2, Psma3, Psma6, Psmb6, Psmc2, Psmc4, Psmc5, Psmd13, Ptbp1, Rab11a, Rab14, Rab5c, Rack1, Rangap1, Rars, Rbbp4, Rbmxl1, Rnh1, Rpl22, Rpl26, Rpl30, Rpl34, Rpl35a, Rpl36-ps3, Rps11, Rps15a, Rps16, Rps19, Rps20, Rps26, Rps27, Rps3a, Rps4x, Rpsa, Rrbp1, Ruvbl1, Sae1, Sept11, Sfpq, Snrpa1, Snrpd3, Snx3, Spata5, Sptbn1, Sri, Stmn1, Syncrip, Tardbp, Tbcb, Tkt, Tln1, Tmpo, Tpm3, Tpm3-rs7, Tpm4, Twf1, Ugdh, Uqcrc1, Uqcrc2, Vars, Vim, Vps35, Yars,Ybx3, Zyx
**invadosome/total proteome ratio ≥ 1,5 :**<br>Acly, Actb, Actg1, Actr3, Anp32b, Anxa2, Arpc4, Atp5a1, Atp5f1, Capg, Capza2, Cct3, Cct5, Cct8, Cdc42, Clta, Copb2, Dars, Dpysl2, Eef1a1, Eef1a2, Eef1d, Eef1g, Eef2, Eif5a, Esd, GAPDH, Glud1, Gnai2, Gps1, Gsn, Hsp90b1, Hspa5, Kif5b, Lrpprc, Msn, Myh11, Myh9, Napa, Nup85, Pdlim1, Pgd, Phgdh, Ppp1ca, Prdx1, Prdx3, Prkaca, Psmb4, Psmc3, Psmd2, Ran, Rpl10, Rpl10a, Rpl12, Rpl23, Rpl23a-ps3, Rpl28, Rpn1, Rps12, Rps13, Rps3, Set, Shmt2, Stip1, Sugt1, Tpm1, Tpm2, Ugp2, Vdac2

Supplementary Table 2: List of proteins identified in invadosomes rosettes and enriched compared to the whole cellular proteome

The proteins are listed using their gene name. The table is subdivided in five categories i) proteins only detected in invadosomes fraction, ii) Invadosome/ total proteome ratio ≥ 10, iii) Invadosome/ total proteome ratio ≥ 5, iv) Invadosome/ total proteome ratio ≥ 2 and v) Invadosome/ total proteome ratio ≥ 1,5.

**Non described in invadosomes but associated to cancer invasion:**<br>Abce1, Acat1, Acly, Actr1a, Ahcy, Ahnak, Anp32a, Anxa2, Bag3, Btf3l4, Cap1, Capg, Caprin1, Cct5, Cct8, Col1a2, Ddx17, Ddx3x, Dpysl2, Eef1a1, Eef1a2, Eif3e, Eif3h, Eif3i, Eif4b, Eif5a, Fhl2, Flnb, Flnc, Fubp1, G3bp1, GAPDH, Gnai2, Hist2h3b, Hnrnpa1, Hnrnpa2b1, Hspa5, Hspa8, Hspd1, Igf2bp1, Igf2bp2, Khsrp, Kpna2, Kpnb1, Lima1, Lrpprc, Map4, Mcm2, Mcm3, Mcm7, Mtco2, Mvp, Myef2, Myof, Ncl, Nedd4l, Nono, Npm1, Numa1, Ogdh, Pa2g4, Pcbp1, Pcbp2, Pdlim1, Phb, Phgdh, Ppp1ca, Prdx1, Prdx2, Prdx3, Prkaca, Psmc2, Ptbp1, Rab21, Rab5c, Ran, Rbbp4, Rpl30, Rpl34, Rps27, Rps3, Ruvbl1, Ruvbl2, Sae1, Sdha, Serbp1, Set, Shmt2, Sri, Stip1, Stmn1, Tkt, Tpm3-rs7, Trim28, Twf1
**Described in invadosomes and associated to cancer invasion:**<br>Actb, Actg1, Actr3, Arpc4, Cald1, Cdc42, Clta, Cnn2, Cnn3, Coro1b, Csrp2, Cttn, Fasn, Flna, Fn1, Gsn, Hnrnpf, Hnrnpk, Hnrnpl, Hnrnpm, Hsp90b1, Igf2bp3, Iqgap1, Kif5b, Lasp1, Msn, Myh9, Plec, Ppp3ca, Rab11a, Rab14, Rack1, Tln1, Tpm1, Tpm2, Tpm3, Vim, Zyx

Supplementary Table 3: **List of proteins identified in invadosomes and enriched compared to the whole cellular proteome already described in the literature in invadosomes or associated to cancer invasion**

The proteins are listed using their gene name.

siRNA name	Target sequence (5’-3’)
si Ctrl	CGTACGCGGAATACTTCGA
si Bag3_1	CCGAAGGGAGGCAGACTCTAA
si Bag3_2	CCCAGGTCAAGTACAAGTCTA
si Ahnak _1	CAGAGGGATGATGGAGTCTTT
si Ahnak_2	TGGCTTGAAGTTGCACCGTAA
si Ckap4_1	CAGGAAGCAGATTAACCTAAA
si Ckap4_2	TAGGTTGTTTCTGAAAGTTGA
si Phb2_1	CTGGATGATGTAGCTATCACA
si Phb2_2	AACGATCGCCACATCACAGAA
si Cpne1_1	CAGGAAAGAAAGACTAGTAAA
si Cpne1_2	CAGGTGATATTTGCAGTGTTA
si Ddx17_1	AGCTACCAATATGATAGGCTA
si Ddx17_2	CCGGACTACTTCTTCAGCCAA
si eEF1A1_2	ACCACCGCTAATTCAAAGCAA
si eEF1A1_3	AAGAACGGTCTCAGAACTGTT
si eIF4A3_1	TGCAGTTGTCTTTCTGCGGAA
si eIF4A3_2	ATGCACATGTACATAATCCGA
si eEF2_1	CCGTGCCATCATGGACAAGAA
si eEF2_2	CAAGCCCGTCCTGATGATGAA
si eIF3H_1	CCAGGACATAATCAAATACAA
si eIF3H_2	CTGGTATCAGTCCACATATTA
si Elavl1_1	ACCAGTTTCAATGGTCATAAA
si Elavl1_2	CACAGTGAAGTTTGCAGCCAA
si eIF3L_1	TACAGGCATATTTACGCCAAA
si eIF3L_2	CTGAAAGGTTCTTCAAGAATA
si Myof_1	AAGGAGGATATTGTACCACAA
si Myof_3	CGGGAAGTCGTTATTGAAATA
si Hnrpl_1	TACGCGTTTAAATGTATTCAA
si Hnrpl_2	CTGCATTTGTCAATTATTCTA
si Pcbp2_1	ACCGACTAATGCCATCTTCAA
si Pcbp2_2	ACCAAAGACTTGACCACTCAA
si Matr3_1	AAGAAGCTTAATTCAAAGAAA
si Matr3_2	TTCCTCATTATCAGAAATTAA
si S100A4_1	CACAGTGCTGAGCAAATTCAA
si S100A4_2	CTGCATTGCCATGATGTGCAA
si Caprin1_1	CAGCACGTCGGGAACAGCTTA
si Caprin1_2	CACAAATGCAAGGGCCCTATA

Supplementary Table 4: List of the different siRNA used in the screening.

Signal processing strategy	Detect LC-MS peaks
Deisotoping mode	Identification based
Use previous peakel detection	false
Signal extraction tolerance	10.0 ppm
Alignment method	ITERATIVE
Max. number of alignment iterations	3
Alignment m/z tolerance	10.0 ppm
Alignment time tolerance (sec)	300
Alignment smoothing method	LANDMARK_RANGE
Alignment window size	50
Alignment window overlap (%)	20
Match between runs m/z tolerance	10.0 ppm
Match between runs time tolerance (sec)	60
Intensity normalization method	INTENSITY_SUM
### POST-PROCESSING PARAMETERS ###	
Use only specific peptides	true
Discard miss cleaved peptides	false
Discard oxidized peptides	false
Apply profile clustering	false
Abundance summarizer method	SUM

Supplementary Table 5: **Parameters used to perform label-free quantification.**

Primary probes	Name	Sequence
	m_beta_actin_01	CTAGAAGCACTTGCGGTGCACGATGGATTACACTCGGACCTCGTCGACATGCATT
	m_beta_actin_02	TGCGCTCAGGAGGAGCAATGATCTTGATTACACTCGGACCTCGTCGACATGCATT
	m_beta_actin_03	TTCATGGTGCTAGGAGCCAGAGCAGTATTACACTCGGACCTCGTCGACATGCATT
	m_beta_actin_04	GCATCGGAACCGCTCGTTGCCAATAGTTACACTCGGACCTCGTCGACATGCATT
	m_beta_actin_05	GCTCATAGCTCTTCTCCAGGGAGGAATTACACTCGGACCTCGTCGACATGCATT
	m_beta_actin_06	CATCTCCTGCTCGAAGTCTAGAGCAACTTACACTCGGACCTCGTCGACATGCATT
	m_beta_actin_07	CGGAGTCCATCACAATGCCTGTGGTATTACACTCGGACCTCGTCGACATGCATT
	m_beta_actin_08	TGAAGGTCTCAAACATGATCTGGGTCATCTTACACTCGGACCTCGTCGACATGCATT
	m_beta_actin_09	CAGTTGGTAACAATGCCATGTTCAATGGGGTTACACTCGGACCTCGTCGACATGCATT
	m_beta_actin_10	CTTGCTGATCCACATCTGCTGGAAGGTTACACTCGGACCTCGTCGACATGCATT
	m_beta_actin_11	CTGTCAGCAATGCCTGGGTACATGGTTTACACTCGGACCTCGTCGACATGCATT
	m_beta_actin_12	CCACCAGACAGCACTGTGTTGGCATATTACACTCGGACCTCGTCGACATGCATT
	m_beta_actin_13	GGTCTTTACGGATGTCAACGTCACACTTCTTACACTCGGACCTCGTCGACATGCATT
	m_beta_actin_14	AGCACAGCTTCTCTTTGATGTCACGCTTACACTCGGACCTCGTCGACATGCATT
	m_beta_actin_15	CATAGCCCTCGTAGATGGGCACAGTGTTACACTCGGACCTCGTCGACATGCATT
	m_beta_actin_16	TGGATGGCTACGTACATGGCTGGGGTTTACACTCGGACCTCGTCGACATGCATT
	m_beta_actin_17	TTCACGGTTGGCCTTAGGGTTCAGGGGTTACACTCGGACCTCGTCGACATGCATT
	m_beta_actin_18	GGGCCACACGCAGCTCATTGTAGAAGTTACACTCGGACCTCGTCGACATGCATT
	m_beta_actin_19	GTGGTGCCAGATCTTCTCCATGTCGTTTACACTCGGACCTCGTCGACATGCATT
	m_beta_actin_20	CTTCAGGGTCAGGATACCTCTCTTGCTTACACTCGGACCTCGTCGACATGCATT
	m_beta_actin_21	TTTGCACATGCCGGAGCCGTTGTCGATTACACTCGGACCTCGTCGACATGCATT
	m_beta_actin_22	GATGGAATTGAATGTAGTTTCATGGATGCCACTTACACTCGGACCTCGTCGACATGCATT
	m_beta_actin_23	GATTCCATACCCAAGAAGGAAGGCTGGAAATTACACTCGGACCTCGTCGACATGCATT
	m_beta_actin_24	GATTTCCCTCTCAGCTGTGGTGGTGATTACACTCGGACCTCGTCGACATGCATT
Secondary probe	Name	Sequence
	FLAP Y-Cy3	/5Cy3/AA TGC ATG TCG ACG AGG TCC GAG TGT AA/3Cy3Sp/

Supplemental Table 6: **List of the probe sequences used for smiFISH**

References

1. Attanasio, F. *et al.* Novel invadopodia components revealed by differential proteomic analysis. *Eur J Cell Biol* **90**, 115-127 (2011).
2. Cervero, P., Himmel, M., Kruger, M. & Linder, S. Proteomic analysis of podosome fractions from macrophages reveals similarities to spreading initiation centres. *Eur J Cell Biol* **91**, 908-922 (2012).
