# Supplementary material for: Combining laser capture microdissection and proteomics reveals an active translation machinery controlling invadosome formation
Source: Nat Commun. 2018 May 23;9:2031. doi: 10.1038/s41467-018-04461-9 (PMC5966458; doi:10.1038/s41467-018-04461-9)
Supplement: Supplementary file 3 — Description of Additional Supplementary Files [file 41467_2018_4461_MOESM3_ESM.pdf]

Description of Additional Supplementary Files

File Name: Supplementary Movie 1
Description: Dynamics of invadosome rosettes in NIH-3T3-Src cells
NIH-3T3-Src cells constitutively expressing the Lifeact-mRuby (red) were analyzed by timelapse microscopy using a video-spinning-disk-FRAP microscope (DMI6000B; Leica). Frames were taken every minute for 30 min.

File Name: Supplementary Data 1
Description: List of proteins identified in invadosomes rosettes and enriched compared to the whole cellular proteome. The Uniprot accession number is indicated, as well as the full name of each protein, the number of specific peptides which allowed the identification and the invadosome /total proteome enrichment ratio. P/A for Present/Absent means detected only in the invadosome sample.

File Name: Supplementary Data 2
Description: List of proteins identified in invadosomes and enriched compared to the whole cellular proteome already described in the literature in invadosomes, involved in matrix degradation or associated to cancer invasion (ND: Non-Determined; PMID: PubMed reference number). Involvement in actin reorganization, cell adhesion, chemotaxis or protein translation is also indicated (yes or ND), this information was extracted from Ingenuity® Pathway Analysis Database (Qiagen).
