# Supplementary material for: Combining laser capture microdissection and proteomics reveals an active translation machinery controlling invadosome formation
Source: Nat Commun. 2018 May 23;9:2031. doi: 10.1038/s41467-018-04461-9 (PMC5966458; doi:10.1038/s41467-018-04461-9)
Supplement: Supplementary file 5 — Supplementary Data 1 [file 41467_2018_4461_MOESM5_ESM.docx]

| **Uniprot**  **accession**  **number** | **gene name** | **description** | **number of specific peptides** | **invadosome/total**  **proteome**  **ratio** |
| --- | --- | --- | --- | --- |
| P61514 | Rpl37a | 60S ribosomal protein L37a | 2 | P/A |
| Q4FK74 | Atp5d | ATP synthase subunit delta, mitochondrial | 2 | P/A |
| Q01149 | Col1a2 | Collagen alpha-2(I) chain | 2 | P/A |
| O88544 | Cops4 | COP9 signalosome complex subunit 4 | 3 | P/A |
| P97310 | Mcm2 | DNA replication licensing factor MCM2 | 2 | P/A |
| Q6A0D1 | Emc2 | ER membrane protein complex subunit 2 | 2 | P/A |
| Q5M9L0 | Eif3h | Eukaryotic translation initiation factor 3 subunit H | 3 | P/A |
| A0A140LJ59 | Eif3k | Eukaryotic translation initiation factor 3 subunit K | 2 | P/A |
| A0A0U1RP81 | Immt | MICOS complex subunit MIC60 | 2 | P/A |
| Q9CZW5 | Tomm70 | Mitochondrial import receptor subunit TOM70 | 2 | P/A |
| Q3U2W2 | Mybbp1a | MYB binding protein (P160) 1a, isoform CRA_b | 3 | P/A |
| Q91VD9 | Ndufs1 | NADH-ubiquinone oxidoreductase 75 kDa subunit, mitochondrial | 2 | P/A |
| E9Q7G0 | Numa1 | Protein Numa1 | 3 | P/A |
| Q9D051 | Pdhb | Pyruvate dehydrogenase E1 component subunit beta, mitochondrial | 2 | P/A |
| P35282 | Rab21 | Ras-related protein Rab-21 | 2 | P/A |
| Q99MR6 | Srrt | Serrate RNA effector molecule homolog | 2 | P/A |
| Q8K2B3 | Sdha | Succinate dehydrogenase [ubiquinone] flavoprotein subunit, mitochondrial | 2 | P/A |
| O35295 | Purb | Transcriptional activator protein Pur-beta | 3 | P/A |
| D3Z0S6 | Snrpa | U1 small nuclear ribonucleoprotein A | 3 | P/A |
| Q91YT7 | Ythdf2 | YTH domain-containing family protein 2 | 2 | P/A |
| B8JJI7 | Mtcl1 | Microtubule cross-linking factor 1 | 2 | 188,77 |
| D3Z5N9 | Gm5449 | MCG49198 | 2 | 47,29 |
| Q99LF4 | Rtcb | tRNA-splicing ligase RtcB homolog | 2 | 22,08 |
| A0A087WR50 | Fn1 | Fibronectin | 26 | 17,69 |
| Q8K4Z5 | Sf3a1 | Splicing factor 3A subunit 1 | 5 | 14,17 |
| Q921M3 | Sf3b3 | Splicing factor 3B subunit 3 | 3 | 12,26 |
| A0A0U1RPL0 | Atxn2l | Ataxin-2-like protein | 2 | 11,14 |
| A0A087WQD6 | Matr3 | Matrin-3 | 3 | 10,91 |
| A0A140LHA2 | Bub3 | Mitotic checkpoint protein BUB3 | 7 | 10,89 |
| E9QL13 | Rbm14 | MCG8382, isoform CRA_c | 3 | 10,77 |
| Q9JLV1 | Bag3 | BAG family molecular chaperone regulator 3 | 3 | 10,64 |
| A2ATP5 | Myef2 | Myelin expression factor 2 | 3 | 10,23 |
| P25206 | Mcm3 | DNA replication licensing factor MCM3 | 4 | 8,89 |
| P62900 | Rpl31 | 60S ribosomal protein L31 | 4 | 8,34 |
| P83882 | Rpl36a | 60S ribosomal protein L36a | 2 | 7,98 |
| O88477 | Igf2bp1 | Insulin-like growth factor 2 mRNA-binding protein 1 | 5 | 7,92 |
| A2A6U3 | Sept9 | Septin-9 | 6 | 7,87 |
| F6XLV1 | Crocc2 | Protein Crocc2 | 2 | 7,84 |
| P60229 | Eif3e | Eukaryotic translation initiation factor 3 subunit E | 10 | 6,87 |
| Q8CGC7 | Eprs | Bifunctional glutamate/proline--tRNA ligase | 9 | 6,86 |
| Q8BJW6 | Eif2a | Eukaryotic translation initiation factor 2A | 3 | 6,52 |
| Q99K48 | Nono | Non-POU domain-containing octamer-binding protein | 5 | 6,47 |
| Q3TXT7 | Ruvbl2 | RuvB-like helicase | 2 | 6,46 |
| A2AFQ2 | Hsd17b10 | 3-hydroxyacyl-CoA dehydrogenase type-2 | 4 | 6,46 |
| P47738 | Aldh2 | Aldehyde dehydrogenase, mitochondrial | 8 | 6,28 |
| A0A0G2JEP0 | Fxr1 | Fragile X mental retardation syndrome-related protein 1 | 4 | 6,23 |
| Q8QZT1 | Acat1 | Acetyl-CoA acetyltransferase, mitochondrial | 4 | 6,19 |
| B2RXM7 | Sarnp | MCG113697 | 4 | 6,15 |
| P70372 | Elavl1 | ELAV-like protein 1 | 5 | 6,05 |
| Q9Z204 | Hnrnpc | Heterogeneous nuclear ribonucleoproteins C1/C2 | 3 | 5,96 |
| A0A0G2JFY5 | Fubp1 | Far upstream element-binding protein 1 | 7 | 5,94 |
| Q9ERG0 | Lima1 | LIM domain and actin-binding protein 1 | 5 | 5,86 |
| P09405 | Ncl | Nucleolin | 18 | 5,83 |
| A0A0N4SUN8 | Serbp1 | Plasminogen activator inhibitor 1 RNA-binding protein | 2 | 5,78 |
| Q9Z2X1 | Hnrnpf | Heterogeneous nuclear ribonucleoprotein F | 9 | 5,71 |
| P62264 | Rps14 | 40S ribosomal protein S14 | 5 | 5,58 |
| B8JK32 | Hnrnpm | Heterogeneous nuclear ribonucleoprotein M | 18 | 5,49 |
| Q00612 | G6pdx | Glucose-6-phosphate 1-dehydrogenase X | 4 | 5,38 |
| Q3V3R1 | Mthfd1l | Monofunctional C1-tetrahydrofolate synthase, mitochondrial | 6 | 5,29 |
| Q62318 | Trim28 | Transcription intermediary factor 1-beta | 7 | 5,22 |
| P48722 | Hspa4l | Heat shock 70 kDa protein 4L | 7 | 5,13 |
| P62192 | Psmc1 | 26S protease regulatory subunit 4 | 4 | 5,13 |
| G3UY38 | Hnrnpl | Heterogeneous nuclear ribonucleoprotein L | 13 | 5,02 |
| P62918 | Rpl8 | 60S ribosomal protein L8 | 3 | 5,00 |
| B1AU75 | Nasp | Nuclear autoantigenic sperm protein | 10 | 4,94 |
| D3Z7P3 | Gls | Glutaminase kidney isoform, mitochondrial | 2 | 4,93 |
| P46061 | Rangap1 | Ran GTPase-activating protein 1 | 6 | 4,91 |
| A0A0B4J1F2 | Map4 | Microtubule-associated protein | 8 | 4,84 |
| B7ZCP4 | Cpne1 | Copine-1 | 4 | 4,82 |
| Q91VM5 | Rbmxl1 | RNA binding motif protein, X-linked-like-1 | 5 | 4,81 |
| Q3U741 | Ddx17 | DEAD (Asp-Glu-Ala-Asp) box polypeptide 17, isoform CRA_a | 3 | 4,79 |
| P31230 | Aimp1 | Aminoacyl tRNA synthase complex-interacting multifunctional protein 1 | 5 | 4,74 |
| A0A0R4J259 | Syncrip | Heterogeneous nuclear ribonucleoprotein Q | 2 | 4,69 |
| O55142 | Rpl35a | 60S ribosomal protein L35a | 3 | 4,68 |
| P60335 | Pcbp1 | Poly(rC)-binding protein 1 | 8 | 4,60 |
| G3UY93 | Vars | Valine--tRNA ligase | 6 | 4,54 |
| Q9JKB3 | Ybx3 | Y-box-binding protein 3 | 3 | 4,54 |
| Q99KP6 | Prpf19 | Pre-mRNA-processing factor 19 | 2 | 4,53 |
| O88569 | Hnrnpa2b1 | Heterogeneous nuclear ribonucleoproteins A2/B1 | 13 | 4,49 |
| P62855 | Rps26 | 40S ribosomal protein S26 | 2 | 4,47 |
| P14733 | Lmnb1 | Lamin-B1 | 7 | 4,45 |
| D3Z1Z8 | Stmn1 | Stathmin | 3 | 4,45 |
| Q3TQX5 | Ddx3x | ATP-dependent RNA helicase DDX3X | 9 | 4,42 |
| E9Q390 | Myof | Myoferlin | 15 | 4,37 |
| P57784 | Snrpa1 | U2 small nuclear ribonucleoprotein A' | 4 | 4,31 |
| B2RTK3 | Hist1h2bm | Histone H2B | 7 | 4,29 |
| P19096 | Fasn | Fatty acid synthase | 21 | 4,27 |
| Q60598 | Cttn | Src substrate cortactin | 16 | 4,19 |
| P48678 | Lmna | Prelamin-A/C | 71 | 4,19 |
| O35286 | Dhx15 | Pre-mRNA-splicing factor ATP-dependent RNA helicase DHX15 | 8 | 4,10 |
| E9Q0U7 | Hsph1 | Heat shock protein 105 kDa | 9 | 4,09 |
| A0A0G2JDW7 | Rps27 | 40S ribosomal protein S27 | 4 | 4,05 |
| Q9DB77 | Uqcrc2 | Cytochrome b-c1 complex subunit 2, mitochondrial | 5 | 4,05 |
| D3YYE1 | Anp32a | Acidic leucine-rich nuclear phosphoprotein 32 family member A | 2 | 4,03 |
| A0A0A6YW67 | Gm8797 | MCG23377, isoform CRA_b | 5 | 4,01 |
| G3XA10 | Gm28062 | Heterogeneous nuclear ribonucleoprotein U, isoform CRA_b | 21 | 3,89 |
| P49722 | Psma2 | Proteasome subunit alpha type-2 | 4 | 3,87 |
| Q61990 | Pcbp2 | Poly(rC)-binding protein 2 | 6 | 3,87 |
| Q99LP6 | Grpel1 | GrpE protein homolog 1, mitochondrial | 2 | 3,81 |
| P61255 | Rpl26 | 60S ribosomal protein L26 | 6 | 3,79 |
| Q9EQK5 | Mvp | Major vault protein | 8 | 3,78 |
| Q8QZY1 | Eif3l | Eukaryotic translation initiation factor 3 subunit L | 9 | 3,72 |
| Q9D1E6 | Tbcb | Tubulin-folding cofactor B | 3 | 3,71 |
| B2M1R6 | Hnrnpk | Heterogeneous nuclear ribonucleoprotein K | 25 | 3,71 |
| A0A087WP83 | Hdlbp | Vigilin | 10 | 3,69 |
| P97855 | G3bp1 | Ras GTPase-activating protein-binding protein 1 | 7 | 3,67 |
| F7CVJ5 | Ahnak2 | Protein Ahnak2 | 10 | 3,66 |
| P97311 | Mcm6 | DNA replication licensing factor MCM6 | 2 | 3,66 |
| E9PYL9 | Gm10036 | Protein Gm10036 | 6 | 3,63 |
| P62196 | Psmc5 | 26S protease regulatory subunit 8 | 3 | 3,60 |
| Q60865 | Caprin1 | Caprin-1 | 2 | 3,59 |
| Q8R1B4 | Eif3c | Eukaryotic translation initiation factor 3 subunit C | 4 | 3,59 |
| A0A0G2JGS4 | Camk2d | Calcium/calmodulin-dependent protein kinase type II subunit delta | 5 | 3,58 |
| Q62261 | Sptbn1 | Spectrin beta chain, non-erythrocytic 1 | 19 | 3,56 |
| A2AVJ7 | Rrbp1 | Ribosome-binding protein 1 | 12 | 3,55 |
| E9PXB7 | Nedd4l | E3 ubiquitin-protein ligase NEDD4-like | 2 | 3,48 |
| P70670 | Naca | Nascent polypeptide-associated complex subunit alpha, muscle-specific form | 5 | 3,47 |
| Q8VIJ6 | Sfpq | Splicing factor, proline- and glutamine-rich | 15 | 3,46 |
| P62889 | Rpl30 | 60S ribosomal protein L30 | 3 | 3,45 |
| E9Q616 | Ahnak | Protein Ahnak | 104 | 3,45 |
| A0A0A6YVV8 | Mbnl1 | Muscleblind-like protein 1 | 2 | 3,42 |
| P61222 | Abce1 | ATP-binding cassette sub-family E member 1 | 3 | 3,40 |
| Q6ZWX6 | Eif2s1 | Eukaryotic translation initiation factor 2 subunit 1 | 4 | 3,36 |
| Q8BGJ5 | Ptbp1 | MCG13402, isoform CRA_a | 18 | 3,35 |
| P60122 | Ruvbl1 | RuvB-like 1 | 5 | 3,34 |
| D3YW87 | Flnc | Filamin-C | 42 | 3,32 |
| O70433 | Fhl2 | Four and a half LIM domains protein 2 | 2 | 3,29 |
| E9Q3W4 | Plec | Plectin | 148 | 3,29 |
| P70168 | Kpnb1 | Importin subunit beta-1 | 12 | 3,29 |
| Q8CGK3 | Lonp1 | Lon protease homolog, mitochondrial | 2 | 3,28 |
| A0A0N4SV66 | H2afj | Histone H2A | 6 | 3,28 |
| Q9CZX8 | Rps19 | 40S ribosomal protein S19 | 4 | 3,26 |
| Q4KL76 | Hspe1 | 10 kDa heat shock protein, mitochondrial | 5 | 3,22 |
| Q80X90 | Flnb | Filamin-B | 36 | 3,22 |
| O35129 | Phb2 | Prohibitin-2 | 9 | 3,21 |
| Q9CZ30 | Ola1 | Obg-like ATPase 1 | 4 | 3,19 |
| P68040 | Rack1 | Receptor of activated protein C kinase 1 | 19 | 3,18 |
| Q61656 | Ddx5 | Probable ATP-dependent RNA helicase DDX5 | 11 | 3,18 |
| P26039 | Tln1 | Talin-1 | 21 | 3,15 |
| B9EI85 | Hist2h3b | Histone H3 | 5 | 3,14 |
| Q9DB20 | Atp5o | ATP synthase subunit O, mitochondrial | 4 | 3,13 |
| P97351 | Rps3a | 40S ribosomal protein S3a | 13 | 3,13 |
| Q6IRU2 | Tpm4 | Tropomyosin alpha-4 chain | 11 | 3,12 |
| P67778 | Phb | Prohibitin | 10 | 3,11 |
| P35278 | Rab5c | Ras-related protein Rab-5C | 7 | 3,09 |
| Q543N3 | Lasp1 | LIM and SH3 domain protein 1 | 9 | 3,07 |
| P38647 | Hspa9 | Stress-70 protein, mitochondrial | 32 | 3,04 |
| Q9D2G2 | Dlst | Dihydrolipoyllysine-residue succinyltransferase component of 2-oxoglutarate dehydrogenase complex, mitochondrial | 2 | 3,03 |
| P20152 | Vim | Vimentin | 110 | 3,02 |
| Q9CZ13 | Uqcrc1 | Cytochrome b-c1 complex subunit 1, mitochondrial | 4 | 3,01 |
| A4FUS1 | Rps16 | 40S ribosomal protein S16 | 5 | 2,99 |
| F8WJ41 | Rps15a | 40S ribosomal protein S15a | 7 | 2,99 |
| A3KFU5 | Pabpc4 | Polyadenylate-binding protein | 4 | 2,99 |
| Q62523 | Zyx | Zyxin | 5 | 2,98 |
| A2RS22 | Coro1b | Coronin | 2 | 2,98 |
| P30416 | Fkbp4 | Peptidyl-prolyl cis-trans isomerase FKBP4 | 4 | 2,98 |
| Q9QZD9 | Eif3i | Eukaryotic translation initiation factor 3 subunit I | 5 | 2,93 |
| Q3U0V1 | Khsrp | Far upstream element-binding protein 2 | 4 | 2,92 |
| E9QA16 | Cald1 | Protein Cald1 | 10 | 2,90 |
| O70435 | Psma3 | Proteasome subunit alpha type-3 | 4 | 2,89 |
| P29341 | Pabpc1 | Polyadenylate-binding protein 1 | 15 | 2,84 |
| Q8BMK4 | Ckap4 | Cytoskeleton-associated protein 4 | 24 | 2,83 |
| P40124 | Cap1 | Adenylyl cyclase-associated protein 1 | 28 | 2,82 |
| A0A087WR97 | Tardbp | TAR DNA-binding protein 43 | 6 | 2,82 |
| B2RT97 | Psmd13 | 26S proteasome non-ATPase regulatory subunit 13 | 4 | 2,79 |
| P46471 | Psmc2 | 26S protease regulatory subunit 7 | 4 | 2,77 |
| O35737 | Hnrnph1 | Heterogeneous nuclear ribonucleoprotein H | 6 | 2,72 |
| Q9JKF1 | Iqgap1 | Ras GTPase-activating-like protein IQGAP1 | 21 | 2,70 |
| Q9D8B3 | Chmp4b | Charged multivesicular body protein 4b | 2 | 2,69 |
| A0A140LIZ5 | Psmc4 | 26S protease regulatory subunit 6B | 3 | 2,68 |
| B7FAU9 | Flna | Filamin, alpha | 89 | 2,67 |
| P49312 | Hnrnpa1 | Heterogeneous nuclear ribonucleoprotein A1 | 12 | 2,66 |
| A2AH85 | Eftud2 | 116 kDa U5 small nuclear ribonucleoprotein component | 4 | 2,66 |
| Q8BGD9 | Eif4b | Eukaryotic translation initiation factor 4B | 3 | 2,65 |
| Q0VDT4 |  | Leucine rich repeat containing 39 | 2 | 2,65 |
| Q9QUM9 | Psma6 | Proteasome subunit alpha type-6 | 6 | 2,64 |
| P63038 | Hspd1 | 60 kDa heat shock protein, mitochondrial | 52 | 2,63 |
| B1AR69 | Myh13 | Protein Myh13 | 3 | 2,62 |
| Q9DCH4 | Eif3f | Eukaryotic translation initiation factor 3 subunit F | 3 | 2,62 |
| B2RXM2 | Gm6793 | EG627828 protein | 12 | 2,62 |
| P24547 | Impdh2 | Inosine-5'-monophosphate dehydrogenase 2 | 3 | 2,62 |
| Q922D8 | Mthfd1 | C-1-tetrahydrofolate synthase, cytoplasmic | 2 | 2,61 |
| Q91YR1 | Twf1 | Twinfilin-1 | 3 | 2,61 |
| D3YX34 | Dctn1 | Dynactin subunit 1 | 2 | 2,59 |
| Q9CPN8 | Igf2bp3 | Insulin-like growth factor 2 mRNA-binding protein 3 | 3 | 2,58 |
| D3Z6Z0 | Snx3 | Sorting nexin-3 | 5 | 2,58 |
| A0A0J9YTY0 | Sept11 | Septin-11 | 3 | 2,57 |
| Q6P069 | Sri | Sorcin | 2 | 2,57 |
| B2RTM0 | Hist2h4 | Histone H4 | 9 | 2,53 |
| P14206 | Rpsa | 40S ribosomal protein SA | 16 | 2,51 |
| Q9CXW3 | Cacybp | Calcyclin-binding protein | 5 | 2,48 |
| Q9ER72 | Cars | Cysteine--tRNA ligase, cytoplasmic | 4 | 2,46 |
| P62814 | Atp6v1b2 | V-type proton ATPase subunit B, brain isoform | 6 | 2,43 |
| D3Z6I8 | Tpm3 | Tropomyosin alpha-3 chain | 3 | 2,42 |
| Q9R1T2 | Sae1 | SUMO-activating enzyme subunit 1 | 4 | 2,41 |
| Q60605 | Myl6 | Myosin light polypeptide 6 | 8 | 2,41 |
| Q5SQB7 | Npm1 | MCG68069 | 14 | 2,40 |
| A0A0G2JDV8 | Cnn3 | Calponin | 3 | 2,40 |
| P62320 | Snrpd3 | Small nuclear ribonucleoprotein Sm D3 | 4 | 2,38 |
| A0A140T8K6 | Rpl36-ps3 | Protein Rpl36-ps3 | 2 | 2,36 |
| D3YVN7 | Gm9755 | Elongation factor Tu | 7 | 2,36 |
| D3YXU1 | Hadhb | Trifunctional enzyme subunit beta, mitochondrial | 3 | 2,33 |
| A0A0A0MQ80 | Spata5 | Spermatogenesis-associated protein 5 | 2 | 2,33 |
| Q3TRJ1 | Vps35 | Vacuolar protein sorting 35, isoform CRA_a | 4 | 2,32 |
| O55137 | Acot1 | Acyl-coenzyme A thioesterase 1 | 3 | 2,31 |
| P62281 | Rps11 | 40S ribosomal protein S11 | 11 | 2,31 |
| A2A7Z4 | Btf3l4 | Transcription factor BTF3 | 2 | 2,30 |
| P52293 | Kpna2 | Importin subunit alpha-1 | 8 | 2,30 |
| P50247 | Ahcy | Adenosylhomocysteinase | 8 | 2,29 |
| P61164 | Actr1a | Alpha-centractin | 7 | 2,29 |
| Q61029 | Tmpo | Lamina-associated polypeptide 2, isoforms beta/delta/epsilon/gamma | 3 | 2,28 |
| P97314 | Csrp2 | Cysteine and glycine-rich protein 2 | 2 | 2,27 |
| Q08093 | Cnn2 | Calponin-2 | 7 | 2,26 |
| Q64521 | Gpd2 | Glycerol-3-phosphate dehydrogenase, mitochondrial | 7 | 2,26 |
| P67984 | Rpl22 | 60S ribosomal protein L22 | 5 | 2,26 |
| Q61171 | Prdx2 | Peroxiredoxin-2 | 8 | 2,25 |
| P40142 | Tkt | Transketolase | 21 | 2,24 |
| Q3U4T8 | Mcm7 | DNA helicase | 2 | 2,24 |
| Q60597 | Ogdh | 2-oxoglutarate dehydrogenase, mitochondrial | 3 | 2,24 |
| A2A7S7 | Yars | Tyrosine--tRNA ligase | 13 | 2,22 |
| Q544H0 | Eif3g | Eukaryotic translation initiation factor 3 subunit G | 4 | 2,22 |
| P63017 | Hspa8 | Heat shock cognate 71 kDa protein | 59 | 2,21 |
| P00405 | Mtco2 | Cytochrome c oxidase subunit 2 | 3 | 2,21 |
| Q60692 | Psmb6 | Proteasome subunit beta type-6 | 3 | 2,20 |
| D3Z2H9 | Tpm3-rs7 | Protein Tpm3-rs7 | 4 | 2,19 |
| Q5XJY5 | Arcn1 | Coatomer subunit delta | 3 | 2,19 |
| P50580 | Pa2g4 | Proliferation-associated protein 2G4 | 13 | 2,15 |
| A6X8Z3 | Igf2bp2 | Insulin-like growth factor 2 mRNA-binding protein 2 | 3 | 2,13 |
| Q9D1R9 | Rpl34 | 60S ribosomal protein L34 | 3 | 2,12 |
| E9PWY9 | Farsa | Phenylalanine--tRNA ligase alpha subunit | 2 | 2,11 |
| P62702 | Rps4x | 40S ribosomal protein S4, X isoform | 11 | 2,10 |
| A0A087WPL5 | Dhx9 | ATP-dependent RNA helicase A | 9 | 2,09 |
| P60867 | Rps20 | 40S ribosomal protein S20 | 3 | 2,06 |
| Q3UD06 | Atp5c1 | ATP synthase subunit gamma | 7 | 2,06 |
| Q50HX4 | Rab14 | RAB14 protein | 3 | 2,05 |
| P63328 | Ppp3ca | Serine/threonine-protein phosphatase 2B catalytic subunit alpha isoform | 2 | 2,04 |
| O70475 | Ugdh | UDP-glucose 6-dehydrogenase | 8 | 2,03 |
| Q9D0I9 | Rars | Arginine--tRNA ligase, cytoplasmic | 16 | 2,03 |
| Q60972 | Rbbp4 | Histone-binding protein RBBP4 | 2 | 2,02 |
| E9Q3P9 | Rab11a | Ras-related protein Rab-11A | 7 | 2,02 |
| Q99JX4 | Eif3m | Eukaryotic translation initiation factor 3 subunit M | 3 | 2,01 |
| Q91VI7 | Rnh1 | Ribonuclease inhibitor | 12 | 2,01 |
| Q9CPS5 | Psmd8 | 26S proteasome non-ATPase regulatory subunit 8 | 2 | 2,00 |
| Q03265 | Atp5a1 | ATP synthase subunit alpha, mitochondrial | 27 | 1,99 |
| P07356 | Anxa2 | Annexin A2 | 33 | 1,96 |
| Q3V117 | Acly | ATP-citrate synthase | 9 | 1,96 |
| P26443 | Glud1 | Glutamate dehydrogenase 1, mitochondrial | 6 | 1,95 |
| B1ATU4 | Gps1 | COP9 signalosome complex subunit 1 | 2 | 1,95 |
| B1AWE0 | Clta | Clathrin light chain A | 4 | 1,95 |
| Q60864 | Stip1 | Stress-induced-phosphoprotein 1 | 18 | 1,95 |
| P62830 | Rpl23 | 60S ribosomal protein L23 | 6 | 1,94 |
| P08752 | Gnai2 | Guanine nucleotide-binding protein G(i) subunit alpha-2 | 6 | 1,94 |
| Q8R480 | Nup85 | Nuclear pore complex protein Nup85 | 2 | 1,94 |
| Q61768 | Kif5b | Kinesin-1 heavy chain | 3 | 1,93 |
| Q5XJF6 | Rpl10a | Ribosomal protein | 4 | 1,93 |
| E9QN08 | Eef1d | Elongation factor 1-delta | 14 | 1,92 |
| Q9EST5 | Anp32b | Acidic leucine-rich nuclear phosphoprotein 32 family member B | 5 | 1,91 |
| B1AXW5 | Prdx1 | Peroxiredoxin-1 | 21 | 1,90 |
| A2AGN7 | Psmc3 | 26S protease regulatory subunit 6A | 4 | 1,88 |
| P62631 | Eef1a2 | Elongation factor 1-alpha 2 | 2 | 1,87 |
| Q9DB05 | Napa | Alpha-soluble NSF attachment protein | 2 | 1,86 |
| P63260 | Actg1 | Actin, cytoplasmic 2 | 10 | 1,84 |
| P20029 | Hspa5 | 78 kDa glucose-regulated protein | 36 | 1,84 |
| B2RRX1 | Actb | Actin, beta | 8 | 1,82 |
| P62827 | Ran | GTP-binding nuclear protein Ran | 10 | 1,81 |
| P10126 | Eef1a1 | Elongation factor 1-alpha 1 | 18 | 1,80 |
| Q5I0W0 | Atp5f1 | ATP synthase F(0) complex subunit B1, mitochondrial | 5 | 1,80 |
| P13020 | Gsn | Gelsolin | 32 | 1,79 |
| P20108 | Prdx3 | Thioredoxin-dependent peroxide reductase, mitochondrial | 2 | 1,78 |
| P05132 | Prkaca | cAMP-dependent protein kinase catalytic subunit alpha | 3 | 1,78 |
| P42932 | Cct8 | T-complex protein 1 subunit theta | 16 | 1,77 |
| Q91YQ5 | Rpn1 | Dolichyl-diphosphooligosaccharide--protein glycosyltransferase subunit 1 | 12 | 1,77 |
| O70400 | Pdlim1 | PDZ and LIM domain protein 1 | 8 | 1,76 |
| P26041 | Msn | Moesin | 21 | 1,76 |
| Q9CZN7 | Shmt2 | Serine hydroxymethyltransferase | 8 | 1,73 |
| P62908 | Rps3 | 40S ribosomal protein S3 | 24 | 1,72 |
| A0A0A0MQM0 | Eif5a | Eukaryotic translation initiation factor 5A | 6 | 1,72 |
| Q9CX34 | Sugt1 | Protein SGT1 homolog | 2 | 1,72 |
| A2AIM4 | Tpm2 | Tropomyosin beta chain | 4 | 1,71 |
| P62137 | Ppp1ca | Serine/threonine-protein phosphatase PP1-alpha catalytic subunit | 4 | 1,71 |
| Q99LB4 | Capg | Capping protein (Actin filament), gelsolin-like | 16 | 1,69 |
| Q6PB66 | Lrpprc | Leucine-rich PPR motif-containing protein, mitochondrial | 5 | 1,68 |
| P63323 | Rps12 | 40S ribosomal protein S12 | 10 | 1,68 |
| P58252 | Eef2 | Elongation factor 2 | 63 | 1,68 |
| Q4FZK2 | Eef1g | Elongation factor 1-gamma | 21 | 1,65 |
| P59999 | Arpc4 | Actin-related protein 2/3 complex subunit 4 | 5 | 1,65 |
| Q3ULF7 | Actr3 | Actin-related protein 3 | 11 | 1,64 |
| E9Q452 | Tpm1 | Tropomyosin alpha-1 chain | 2 | 1,62 |
| E9QPE7 | Myh11 | Myosin-11 | 2 | 1,62 |
| Q61753 | Phgdh | D-3-phosphoglycerate dehydrogenase | 16 | 1,61 |
| O55029 | Copb2 | Coatomer subunit beta' | 5 | 1,60 |
| O08553 | Dpysl2 | Dihydropyrimidinase-related protein 2 | 11 | 1,60 |
| P62301 | Rps13 | 40S ribosomal protein S13 | 6 | 1,60 |
| H3BJL6 | Esd | S-formylglutathione hydrolase | 14 | 1,59 |
| P41105 | Rpl28 | 60S ribosomal protein L28 | 4 | 1,58 |
| I7HLV2 | Rpl10 | 60S ribosomal protein L10 | 6 | 1,58 |
| P99026 | Psmb4 | Proteasome subunit beta type-4 | 7 | 1,58 |
| Q91ZJ5 | Ugp2 | UTP--glucose-1-phosphate uridylyltransferase | 2 | 1,57 |
| A0A0N4SVM0 | Capza2 | F-actin-capping protein subunit alpha-2 | 3 | 1,56 |
| P80316 | Cct5 | T-complex protein 1 subunit epsilon | 12 | 1,56 |
| A2BE93 | Set | Protein SET | 4 | 1,55 |
| P08113 | Hsp90b1 | Endoplasmin | 35 | 1,54 |
| P80318 | Cct3 | T-complex protein 1 subunit gamma | 13 | 1,53 |
| Q9DCD0 | Pgd | 6-phosphogluconate dehydrogenase, decarboxylating | 14 | 1,53 |
| Q3TKV1 | Psmd2 | 26S proteasome non-ATPase regulatory subunit 2 | 9 | 1,53 |
| P60766 | Cdc42 | Cell division control protein 42 homolog | 2 | 1,52 |
| D2KHZ9 | GAPDH | Glyceraldehyde-3-phosphate dehydrogenase | 51 | 1,52 |
| A0A140T8M7 | Rpl23a-ps3 | Protein Rpl23a-ps3 | 5 | 1,52 |
| G3UX26 | Vdac2 | Voltage-dependent anion-selective channel protein 2 | 4 | 1,51 |
| Q922B2 | Dars | Aspartate--tRNA ligase, cytoplasmic | 9 | 1,51 |
| Q8VDD5 | Myh9 | Myosin-9 | 116 | 1,51 |
| P35979 | Rpl12 | 60S ribosomal protein L12 | 7 | 1,50 |
