# Supplementary material for: Combining laser capture microdissection and proteomics reveals an active translation machinery controlling invadosome formation
Source: Nat Commun. 2018 May 23;9:2031. doi: 10.1038/s41467-018-04461-9 (PMC5966458; doi:10.1038/s41467-018-04461-9)
Supplement: Supplementary file 4 [file 41467_2018_4461_MOESM6_ESM.docx]

| gene name | description | Described in invadosomes | Associated to cancer invasion | matrix degradation | Actin reorganisation | Cell adhesion | Chemotaxis | Translation |
|---|---|---|---|---|---|---|---|---|
| Abce1 | ATP-binding cassette sub-family E member 1 | ND | yes PMID:27314749 | ND | | | | |
| Acat1 | Acetyl-CoA acetyltransferase, mitochondrial | ND | yes PMID:27177773 | ND | | | | |
| Acly | ATP-citrate synthase | ND | yes PMID:19795461; PMID:26928812 | ND | | | | |
| Actb | Actin, beta | yes | yes PMID:26008973 | yes | | yes | | |
| Actg1 | Actin, cytoplasmic 2 | yes | yes PMID:24284654 | yes | | | | |
| Actr1a | Alpha-centractin | ND | yes for beta-centractin PMID:17619203 | ND | | | | |
| Actr3 | Actin-related protein 3 | yes PMID:15093736; PMID:24290759; PMID:17483334 | yes PMID:18751399 | yes PMID: 2002374[illegible] | | yes | | |
| Ahcy | Adenosylhomocysteinase | ND | yes PMID:23353701 | ND | | | | |
| Ahnak | Protein Ahnak | ND | yes PMID:24253341 | ND | | | | |
| Anp32a | Acidic leucine-rich nuclear phosphoprotein 32 family memb | ND | yes PMID:26918356 | ND | | | | |
| Anxa2 | Annexin A2 | ND | yes PMID:25697644; PMID:20383413; PMID:19585 | PMID: 26438086 | | yes | yes | |
| Arpc4 | Actin-related protein 2/3 complex subunit 4 | yes PMID:25824735 | yes PMID:26648486 | ND | | | | |
| Bag3 | BAG family molecular chaperone regulator 3 | ND | yes PMID:17974966 | ND | | | | |
| Btf3l4 | Transcription factor BTF3 | ND | yes PMID:28276310 | ND | | | | |
| Cald1 | Protein Cald1 | yes PMID:16595550; PMID:8505382; PMID:17224451; PMID:17239373; PMID:19349302; PMID:20733006; PMID:17631293 | yes but negative PMID:17631293; PMID:23265641 | ND | | yes | | |
| Cap1 | Adenylyl cyclase-associated protein 1 | ND | yes PMID:26810579 | ND | yes | | | |
| Capg | Capping protein (Actin filament), gelsolin-like | No PMID:24236012 | yes PMID:23085225; PMID:22155129 | No PMID: 24236012 | | | yes | |
| Caprin1 | Caprin-1 | ND | yes PMID:23953883 | ND | | yes | | yes |
| Cct5 | T-complex protein 1 subunit epsilon | ND | yes PMID:20442418 | ND | | | | |
| Cct8 | T-complex protein 1 subunit theta | ND | yes PMID:26304164 | ND | | | | |
| Cdc42 | Cell division control protein 42 homolog | yes PMID:16546575; PMID:25777055 | yes PMID:22879067; PMID:18835169; PMID:23884 | yes PMID: 2937455[illegible] | yes | yes | yes | |
| Clta | Clathrin light chain A | yes PMID:18799755; PMID:1706278; PMID:23076136; PMID:22564726 | yes PMID:17545607; PMID:23076136 | ND | | | | |
| Cnn2 | Calponin-2 | yes PMID:16546560 | yes but negative PMID:16271067 | ND | | | | |
| Cnn3 | Calponin | yes PMID:12808045; PMID:14676275 PMID:16546560 | yes PMID:16271067 | ND | | | | |
| Col1a2 | Collagen alpha-2(I) chain | ND | yes PMID:15059891 | ND | | | | |
| Coro1b | Coronin | yes PMID:10328951; PMID:23410663 | yes PMID:24918434 | ND | yes | | yes | |
| Csrp2 | Cysteine and glycine-rich protein 2 | yes PMID:26883198 | yes PMID:26883198 | ND | | yes | | |
| Cttn | Src substrate cortactin | yes PMID:28287395 | yes PMID:22566665 | Yes PMID: 2937455[illegible] | yes | yes | | |
| Ddx17 | DEAD (Asp-Glu-Ala-Asp) box polypeptide 17, isoform CRA_a | ND | yes PMID:22266867 | ND | | | | |
| Ddx3x | ATP-dependent RNA helicase DDX3X | ND | yes PMID:26892600 | ND | | | | yes |
| Dpysl2 | Dihydropyrimidinase-related protein 2 | ND | yes PMID:24036111 | ND | yes | | | |
| Eef1a1 | Elongation factor 1-alpha 1 | ND | yes PMID:28393218 | ND | | | | yes |
| Eef1a2 | Elongation factor 1-alpha 2 | ND | yes PMID:19138673; PMID:23739844; | ND | | yes | | yes |
| Eif3e | Eukaryotic translation initiation factor 3 subunit E | ND | yes PMID:22907435 | ND | | | | yes |
| Eif3h | Eukaryotic translation initiation factor 3 subunit H | ND | yes PMID:27340783 | ND | | | | yes |
| Eif3i | Eukaryotic translation initiation factor 3 subunit I | ND | yes PMID:25609201 | ND | | | | yes |
| Eif4b | Eukaryotic translation initiation factor 4B | ND | yes PMID:25955794 | ND | | | | yes |
| Eif5a | Eukaryotic translation initiation factor 5A | ND | yes PMID:23733422; PMID:20830705; PMID:26282 | ND | | | | yes |
| Fasn | Fatty acid synthase | yes PMID:22238651 | yes PMID:2663225 | ND | | yes | | |
| Fhl2 | Four and a half LIM domains protein 2 | ND | yes PMID:27892920 | ND | | | | |
| Flna | Filamin, alpha | yes PMID:22334688; PMID:16461769; PMID:19656240; PMID:23632887; PMID:24821726; PMID:25481874 | yes but for filamin C PMID:28031525 | yes PMID: 2259552[illegible] | yes | yes | | |

| | | | | | | | | |
|---|---|---|---|---|---|---|---|---|
| Flnb | Filamin-B | ND | yes PMID:25925610 | yes PMID: 2002374 | yes | yes | | |
| Flnc | Filamin-C | ND | yes PMID:28031525 | ND | | | | |
| Fn1 | Fibronectin | yes PMID:20656375 | yes PMID:6324988 | yes PMID: 8106567 | yes | yes | yes | yes |
| Fubp1 | Far upstream element-binding protein 1 | ND | yes PMID:26469968 | ND | | | | |
| G3bp1 | Ras GTPase-activating protein-binding protein 1 | ND | yes PMID:25809930 | ND | | | | |
| GAPDH | Glyceraldehyde-3-phosphate dehydrogenase | ND | yes PMID:25944651 | ND | | | | |
| Gnai2 | Guanine nucleotide-binding protein G(i) subunit alpha-2 | ND | yes PMID:20054866 | ND | | yes | yes | |
| Gsn | Gelsolin | yes PMID:16546574 | yes PMID:22927998;PMID:26662962; PMID:2614 | ND | yes | yes | yes | |
| Hist2h3b | Histone H3 | ND | yes PMID:23557258 | ND | | | | |
| Hnrnpa1 | Heterogeneous nuclear ribonucleoprotein A1 | ND | yes PMID:22821376 | ND | | | | |
| Hnrnpa2b1 | Heterogeneous nuclear ribonucleoproteins A2/B1 | ND | yes PMID:24638979 | ND | | | | |
| Hnrnpf | Heterogeneous nuclear ribonucleoprotein F | yes PMID:22721921 | ND | ND | | | | |
| Hnrnpk | Heterogeneous nuclear ribonucleoprotein K | yes PMID:22721921 | yes PMID:28423622 | ND | | | | yes |
| Hnrnpl | Heterogeneous nuclear ribonucleoprotein L | yes PMID:22721921 | yes PMID:28088793 | ND | | | | |
| Hnrnpm | Heterogeneous nuclear ribonucleoprotein M | yes PMID:22721921 | yes PMID:28393427 | ND | | | | |
| Hsp90b1 | Endoplasmin | yes PMID:21323861 | yes PMID:18482745; PMID:25841765; PMID:26108 | ND | | | | |
| Hspa5 | 78 kDa glucose-regulated protein | ND | yes PMID:25218495; PMID:20082722; PMID:22546 | ND | | | | yes |
| Hspa8 | Heat shock cognate 71 kDa protein | ND | yes PMID:25527454; PMID:27334118 | ND | | | | |
| Hspd1 | 60 kDa heat shock protein, mitochondrial | ND | yes PMID:25207654 | ND | | | yes | |
| Igf2bp1 | Insulin-like growth factor 2 mRNA-binding protein 1 | ND | yes PMID:24632613 | ND | | | yes | yes |
| Igf2bp2 | Insulin-like growth factor 2 mRNA-binding protein 2 | ND | yes PMID:27733218 | ND | | | | yes |
| Igf2bp3 | Insulin-like growth factor 2 mRNA-binding protein 3 | yes PMID:22052854; PMID:22859271 | yes PMID:23647076 | ND | | | | yes |
| Iqgap1 | Ras GTPase-activating-like protein IQGAP1 | yes PMID:24821726; PMID:27269065; PMID:18541705; PMID:28098764 | yes PMID:19856315 | yes PMID: 1854170 | | yes | | |
| Khsrp | Far upstream element-binding protein 2 | ND | yes PMID:28275056 | ND | | | | |
| Kif5b | Kinesin-1 heavy chain | yes PMID:20505159 | yes PMID:26002460 | ND | | | | |
| Kpna2 | Importin subunit alpha-1 | ND | yes PMID:27078844 | ND | | yes | | |
| Kpnb1 | Importin subunit beta-1 | ND | yes PMID:28427184 | ND | | | | |
| Lasp1 | LIM and SH3 domain protein 1 | yes PMID:22514729; PMID:27588391 | yes PMID:23254782 | yes PMID: 27588391 | | | | |
| Lima1 | LIM domain and actin-binding protein 1 | ND | yes PMID:28093207 | ND | | yes | | |
| Lrpprc | Leucine-rich PPR motif-containing protein, mitochondrial | ND | yes PMID:22326293 | ND | | | | |
| Map4 | Microtubule-associated protein | ND | yes PMID:26876215 | ND | | | | |
| Mcm2 | DNA replication licensing factor MCM2 | ND | yes PMID:20661220 | ND | | | | |
| Mcm3 | DNA replication licensing factor MCM3 | ND | yes PMID:20661220 | ND | | | | |
| Mcm7 | DNA helicase | ND | yes PMID:26622601; PMID:28432562; PMID:16247 | ND | | | | |
| Msn | Moesin | yes PMID:24891603; PMID:24127566 | yes PMID:28476784 ; PMID:25299115; PMID:1972 | yes PMID: 2489160 | yes | yes | | |
| Mtco2 | Cytochrome c oxidase subunit 2 | ND | yes PMID:19289149 | ND | | | | |
| Mvp | Major vault protein | ND | yes PMID:24243798 | ND | | | | |
| Myef2 | Myelin expression factor 2 | ND | yes Chung et al, Int J Clin Exp Pathol 2017; 10(4): | ND | | | | |
| Myh9 | Myosin-9 | yes PMID:23361003; PMID:18718759 | yes PMID:25826333; PMID:27262074; PMID:21245 | ND | | | | |
| Myof | Myoferlin | ND | yes PMID:22135466 | ND | | yes | | |
| Ncl | Nucleolin | ND | yes PMID:25866190 | ND | | | | yes |
| Nedd4l | E3 ubiquitin-protein ligase NEDD4-like | ND | yes PMID:21039987 | ND | | | | |
| Nono | Non-POU domain-containing octamer-binding protein | ND | yes PMID:27259250 | ND | | | | |
| Npm1 | MCG68069 | ND | yes PMID:24796332 | ND | | | | |
| Numa1 | Protein Numa1 | ND | yes PMID:22719996 | ND | | | | |
| Ogdh | 2-oxoglutarate dehydrogenase, mitochondrial | ND | yes PMID:23152800 | ND | | | | |
| Pa2g4 | Proliferation-associated protein 2G4 | ND | yes PMID:25917452 | ND | | | | |
| Pcbp1 | Poly(rC)-binding protein 1 | ND | yes PMID:27683057 | ND | | | | yes |
| Pcbp2 | Poly(rC)-binding protein 2 | ND | yes PMID:26761212 | ND | | | | |

| | | | | | | | | |
|---|---|---|---|---|---|---|---|---|
| Pdlim1 | PDZ and LIM domain protein 1 | ND | yes PMID:24662836; PMID:26119933 | ND | | | | |
| Phb | Prohibitin | ND | yes PMID:25575814 | ND | | | | |
| Phgdh | D-3-phosphoglycerate dehydrogenase | ND | yes PMID:23229761 | ND | | | | |
| Plec | Plectin | yes PMID:18553359; PMID:19656240; PMID:21821021; PMID:24810881 | yes PMID:22245045 | ND | | yes | yes | |
| Ppp1ca | Serine/threonine-protein phosphatase PP1-alpha catalytic | ND | yes PMID:21847129; PMID:25240408 | ND | | | | yes |
| Ppp3ca | Serine/threonine-protein phosphatase 2B catalytic subunit | yes PMID:12631724 | yes PMID:20422345 | ND | | | | |
| Prdx1 | Peroxiredoxin-1 | ND | yes PMID:23065574 ; PMID:24297309; PMID:2661 | ND | | | | |
| Prdx2 | Peroxiredoxin-2 | ND | yes PMID:24185040 | ND | | yes | | |
| Prdx3 | Thioredoxin-dependent peroxide reductase, mitochondrial | ND | yes but negative PMID:26983019 | ND | | | | |
| Prkaca | cAMP-dependent protein kinase catalytic subunit alpha | ND | yes PMID:22954688 | ND | | yes | | |
| Psmc2 | 26S protease regulatory subunit 7 | ND | yes PMID:27888613 | ND | | | | |
| Ptbp1 | MCG13402, isoform CRA_a | ND | yes PMID:28404950 | ND | | yes | | yes |
| Rab11a | Ras-related protein Rab-11A | yes PMID:24302731 | yes PMID:28057129; PMID:15805276 | ND | | yes | | |
| Rab14 | RAB14 protein | yes PMID:23606746 | yes PMID:27994670 | ND | | | | |
| Rab21 | Ras-related protein Rab-21 | ND | yes PMID:19953096 | ND | | yes | | |
| Rab5c | Ras-related protein Rab-5C | ND | yes PMID:22734003 | ND | | | | |
| Rack1 | Receptor of activated protein C kinase 1 | yes PMID:15178345 | yes PMID:23912224 | ND | | yes | | yes |
| Ran | GTP-binding nuclear protein Ran | ND | yes PMID:18794800 | ND | yes | | | |
| Rbbp4 | Histone-binding protein RBBP4 | ND | yes PMID:26607076 | ND | yes | yes | | |
| Rpl30 | 60S ribosomal protein L30 | ND | yes PMID:27259250 | ND | | | | yes |
| Rpl34 | 60S ribosomal protein L34 | ND | yes PMID:28109079 | ND | | | | |
| Rps27 | 40S ribosomal protein S27 | ND | yes PMID:23803695 | ND | | | | |
| Rps3 | 40S ribosomal protein S3 | ND | yes PMID:25449781 | ND | | | | yes |
| Ruvbl1 | RuvB-like 1 | ND | yes PMID:24728183 | ND | | | | |
| Ruvbl2 | RuvB-like helicase | ND | yes PMID:22341977 | ND | | | | |
| Sae1 | SUMO-activating enzyme subunit 1 | ND | yes PMID:25628926 | ND | | | | |
| Sdha | Succinate dehydrogenase [ubiquinone] flavoprotein subuni | ND | yes PMID:28500238 | ND | | | | |
| Serbp1 | Plasminogen activator inhibitor 1 RNA-binding protein | ND | yes PMID:25359494 | ND | | | | |
| Set | Protein SET | ND | yes PMID:25234598 | ND | | | | |
| Shmt2 | Serine hydroxymethyltransferase | ND | yes PMID:27666119 | ND | | | | |
| Sri | Sorcin | ND | yes PMID:25567655 | ND | | | | |
| Stip1 | Stress-induced-phosphoprotein 1 | ND | yes PMID:24488757 | ND | | yes | | |
| Stmn1 | Stathmin | ND | yes PMID:27806343 | ND | | yes | | |
| Tkt | Transketolase | ND | yes PMID:26406948 PMID:27698919 | ND | | | | |
| Tln1 | Talin-1 | yes PMID:24821726 | yes PMID:20160039 | yes PMID: 2489160 | yes | yes | | |
| Tpm1 | Tropomyosin alpha-1 chain | yes PMID:16546574 | yes PMID:20971066 ; PMID:28276320 | ND | | yes | | |
| Tpm2 | Tropomyosin beta chain | yes PMID:16546574; PMID:23302954 | yes PMID:24853179 | ND | | yes | | |
| Tpm3 | Tropomyosin alpha-3 chain | yes PMID:8505382; PMID:14676275; PMID:16765662; PMID:17224451 | yes PMID:24913705 | ND | | yes | | |
| Tpm3-rs7 | Protein Tpm3-rs7 | ND | yes PMID:24913705 | ND | | | | |
| Trim28 | Transcription intermediary factor 1-beta | ND | yes PMID:27412325 | ND | | | | |
| Twf1 | Twinfilin-1 | ND | yes PMID:23224145 | ND | | | | |
| Vim | Vimentin | yes PMID:10459017; PMID:18087726; PMID:21969470; PMID:23178938; PMID:20421424; PMID:23786849; PMID:24810881; PMID:26466680 | yes PMID:18790770 | ND | | yes | | |
| Zyx | Zyxin | yes PMID:14625385; PMID:15093736; PMID:22514729; PMID:23361003 | yes PMID:23292068 | ND | | yes | | |
