# Supplementary material for: Combining laser capture microdissection and proteomics reveals an active translation machinery controlling invadosome formation
Source: Nat Commun. 2018 May 23;9:2031. doi: 10.1038/s41467-018-04461-9 (PMC5966458; doi:10.1038/s41467-018-04461-9)
Supplement: Supplementary file 2 — Peer Review File [file 41467_2018_4461_MOESM2_ESM.pdf]

Reviewers' comments:

Reviewer #1 (Remarks to the Author):

The authors describe a proteomics analysis of the invadosome of PFA fixed NIH-3T3-Src cells, which have been grown in SILAC media to differentiate proteins from the NIH-3T3-Src cells from back ground. Further validation and functional characterization of the proteins was then performed using siRNA techniques. An intriguing result is that invadosomes possess their own translation activity, consistent with other subcellular compartments.

One important question not adequately addressed here is if the large rosettes of invadosomes of the NIH-3T3-Src cells, and which is crucial to the success of the reported experiments, are typical of non-rosette invadosomes. The different structure of the rosettes suggests not, and so it is uncertain how the main conclusions drawn from this work translates to patient tumor cells.

An important novel aspect of the work repeatedly claimed by the authors is the automation of LCM to isolate a sufficient number of invadosomes. However, while very useful this reviewer is not convinced by the novelty; LCM has been previously directed by other imaging modalities, followed by proteomics, e.g. for IR microscopy (see Großerueschkamp et al. Scientific Reports 7:44829, 2017) and even imaging mass spectrometry (Dilillo et al. Journal of Proteome Research, 16(8):2993-3001, 2017). Furthermore the throughput claimed by the authors, isolation of 900 rosettes per hour, equates to one isolation every 4 seconds. This is to short for any form of manual QC and no automated QC is described. For example Figure 1a shows that the invadosomes can also reside below the cell body and close to the nucleus, and the invadosomes can reside close together (and thus would be merged by the “morphological

closing and hole filling performed during the segmentation algorithm”). In this reviewer’s opinion the authors have not demonstrated how they avoid/minimize the contribution from non-invadosome structures. At the very least the authors should provide the ImageJ code so that the work may be reproduced and confirmed (it is all very good that they claim they can do it but the reader is not able to check the ImageJ code to check the accuracy of the workflow). Without adding the code itself as supplementary information the impact of the work is greatly reduced.

Statistics - while it may be argued that the low throughput of the invadosome isolation makes any statistical analysis difficult (because repeats are very long, involved experiments), the independent functional validation appears strong. In this reviewer's mind, independent validation is always superior adn should be commended.

Regarding the protein quantitation dilution series - This reviewer is not convinced with this methodology - primarily because the proteome of the invadosome is much smaller than that of the NIH-3T3-Src cells used for the dilution curve - accordingly one cannot assume an equal mass spectrometric response of both proteomes.

The work is based on cell lines that exhibit large invadosome rosettes that are labeled, thus enabling their localization for LCM. However it would be enormously of interest to the reader if the authors could expand on how this technology could be developed further to investigate the proteome of invadosomes from patient tumor cells, to investigate if the invadosome proteome changes with the invasiveness of the tumor cells.

Subject to these caveats, this review recommends major revision

Reviewer #2 (Remarks to the Author):

Saltel and all have an interesting approach to address the invadosome proteome. Although they here focus on the generation mechanism of the functional proteins by local translational control tat likely regulate invadosome structure and function.

Major comments:

- Confocal images (or image stacjks) increase information in Z. DO NOT USE max, intensity projection in any of the colocalization studies. Show the Z-stacks (Suppl?)

- Always show the non-merged channels in B/W (Suppl).

- Fig.1a. Unclear because lack of control (Src RNAi).

- Fig.1b. Why not show image of cells after microdissection, incl. counterstain with e.g. Hoechst?

- Fig.1c. Discuss high amount of contaminating peptides.

- Fig.3. Should NOT BE MAX PROJECTIONS

- Fig.3b. Use B/W images for seperate channels. Discuss why some proteins of interest are present at some invadosomes but not others. Ab specificity is questionable. Use RNAi against eEF2, Caprin to addres specificity (or CHX).

- Fig.3c. Insets are not informative at this size.

- Fig.4b. CLEM is ok. Discuss lack of Z-correlation(always a problem with CLEM).

- Fig.4. Despite proper fixation protocols, ultrastructure is hampered. Why? Discuss.

- Fig.4. How are the authors sure that the small dots are ribosomes? Quite electron dense compared to the typical ribosome appearance in EM.

Minor comments:

- Homemade ImageJ tools are used: Make these public in the suppl. material.

- Nomenclature initiation factors is inconsistent (sometimes wrong) eIF3 (Not EIF3)

- line 645 : Surprising high amount of contaminating peptides. Please discuss.

- too many mentions of 'we .... for the first time...'

- line 576: EM images were stacked. Please explain.

Reviewer #3 (Remarks to the Author):

The authors employ Laser Microdissection of mRuby-LifeAct labeled fixed cell protrusion rosettes to study the proteome of the protrusions as “invadosomes”. The claimed novel finding is the authors identification of a large number of proteins involved in mRNA translation. The authors conclude that “invadasomes concentrate mRNA and exhibit their own local translation activity. A notable aspect of the manuscript is the development of automated image analysis method to reduce the considerable time of the microscopic operator to conduct the microdissection of large number of labeled cell protrusions for the MS analysis. The technical methods are exciting and presented well, and they should be of broad interest. Unfortunately the new knowledge gained about invadosome function, compared to past work, is very limited as delineated below.

1. The authors should make a list of the functionally important categories of known proteins known to be within invadapodia involved in matrix degradation, chemotactic sensing, actin protrusion regulation, adhesion, de adhesion and transcriptome regulation. Under each category they should list whether or not they found this molecule enriched in the invadosomes by MS compared to the non invadosome cell body. Many of the expected proteins are missing from their MS list. Typical invadosome markers well established to be enriched within invadosomes include actin-regulatory proteins such as the Arp2/3 complex, cortactin and WASP or N-WASP (Guirguis R. et al 1987, Linder and Aepfelbacher, 2003, 2009; Buccione et al., 2004, and 2009; Gimona and Buccione, 2006; Weaver, 2006; which colocalize with the actin- rich core of both structures. Functionally invadosomes are regulated by tyrosine kinase signaling, corresponding to a high local enrichment of phosphotyrosine residues (Linder and Aepfelbacher, 2003; Luxenburg et al., 2006). Invadosomes are also enriched in matrix metalloproteinases, both membrane associated and secreted latent enzymes (Journal of Cell Science 122, 3009-3013), integrins, growth factor chemotactic receptors, and focal adhesion signaling proteins such as RhoA-Rho-kinase.

2. The authors fail to find by mass spec (MS) many of the expected MMPs, TIMPs, Integrins, Focal Adhesion and TK proteins confirmed to be involved in the function of invadopodia. The finding of mRNA processing proteins by MS compared to these other expected proteins may be simply due to their much higher relative abundance compared to the other invadosome molecular machinery, and the low sensitivity of the MS methods used.

3. The title is somewhat misleading since the manuscript is a proteomic based analysis of invadosomes, and not a discovery of the mRNA species that are selectively translated at the invadosome body.

4. Even though the authors show by several independent methods that proteins involved in mRNA translation and that RNA is localized in the invadosome they don’t take this finding further to show its mechanistic role. The manuscript and does not discover novel specific mRNA species for proteins specifically involved in functional aspects of invadosome function. What specific proteins are being locally translated in the invadosome and why is this important for the invadosome function?

5. More information should be provided describing the automation method so it can be used and reproduced by others. How will the authors make it available?

Minor comment. The authors do not review invadosome or pseudopod shaving after cytokine induction of invasion as previous means to enrich invadosomes (Guirguis R. 1987 Science), or adequately review previous algorithms for pseudopod tracking Bosgraaf et al. This would round out the review of past literature.

Reviewer #4 (Remarks to the Author):

In this study, Ezzoukhry et al, developed a pipeline that combines laser micro-dissection and mass spectrometry to analyze the molecular composition of cellular structures called invadodosmes. Besides classical invadosome markers, they found specific components of translational machinery, suggesting that invadosomes represents sites of active protein translation.

The study will be of interest to a broad community because of the unique combination of methods that allows studying molecular composition of subcellular structures. As it require only small amount of material, it will also be a powerful method to identify protein signatures of specific regions of tissues. Besides methodological advances, the study also provides novel findings. Beautiful correlative light and electron microscopy and functional assays convincingly show that there is an active protein translation at invadosomes. Data are clear and convincing and statistical methods are appropriate.

I only have few concerns. The whole study is done using fibroblasts overexpressing Src that develop numerous and well-defined rosettes. As fibroblasts are not invasive cells per se, it is important to validate the study, or at least the major findings, in intrinsically invading cells such as cancer cells or osteoclasts. Proteomic study of cancer cells would be difficult as invadopodia are much smaller structures, but localization study of eEF1A1, eEF2 and caprin1, and eventually depletion assays, could be performed.

Minor points:

1. Fig. 1a. It would be s to show that degradation of the matrix is specifically occurring at rosettes. Thus, co-localization of invadosomes and gelatin degradation foci should be shown.

2. Fig 3e. it would be better to split channels and presented them as black/white images in order to appreciate enrichment of eEF1A1, eEF2 and caprin1 in rosettes relative to the rest of the cell.

3. Data presented at Fig 3e should be quantified:

- enrichment of eEF1A1, eEF2 and caprin1 in rosettes relative to the rest of the cell
- Proportion of rosettes containing eEF1A1, eEF2 and caprin1
- If eEF1A1, eEF2 and caprin1 are not present in all rosettes, it would be interesting to investigate if they are associated with newly forming or actively degrading rosettes.

4. While degradation capacity of cells was reduced upon depletion caprin1, there were no effect on invadosomes formation. The authors should at least discuss what could be the underlying mechanism.

Point-by-point response to the reviewer comments

All the changes in the text are in red. We adapted the text of the manuscript as follows:

Reviewer #1 (Remarks to the Author):

The authors describe a proteomics analysis of the invadosome of PFA fixed NIH-3T3-Src cells, which have been grown in SILAC mdia to differentiate proteins from the NIH-3T3-Src cells from back ground. Further validation and functional characterization of the proteins was then performed using siRNA techniques. An intriguing result is that invadosomes possess their own translation activity, consistent with other subcellular compartments.

One important question not adequately addressed here is if the large rosettes of invadosomes of the NIH-3T3-Src cells, and which is crucial to the success of the reported experiments, are typical of non-rosette invadosomes. The different structure of the rosettes suggests not, and so it is uncertain how the main conclusions drawn from this work translates to patient tumor cells.

Indeed, we realized this proof of concept using invadosomes rosettes from NIH-3T3-Src cells that correspond to large structures. This model is widely used to study invadosomes. Moreover, and as suggested by the reviewer 4, we tested our data on other invadosome structures and we confirmed that depletion of translation factors decrease tumor cell ability to form invadosomes (Fig. S6)

About the translation to patient tumor cells, we already adapted this methodology to fixed tissues (Henriet et al, Hepatology 2017). However, even if we defined that the minimal area possible to microdissect with our system is around 1 μm^2, for the moment we are not at the subcellular level within tissues. Thus, we agree with the reviewer and this is why we modulated our conclusion accordingly (page 19).

An important novel aspect of the work repeatedly claimed by the authors is the automation of LCM to isolate a sufficient number of invadosomes. However, while very useful this reviewer is not convinced by the novelty; LCM has been previously directed by other imaging modalities, followed by proteomics, e.g. for IR microscopy (see Großerueschkamp et al. Scientific Reports 7:44829, 2017) and even imaging mass spectrometry (Dilillo et al. Journal of Proteome Research, 16(8):2993-3001, 2017).

Indeed, LCM has already been associated with proteomic analyses, but it's the first study that uses this combination for subcellular proteomics analyses. As mentioned in discussion (page 16, line 324), the only comparable approach is the proteome study of Lewy bodies that are similar in size to one or several cells. As mentioned by Dilillo et al. cited by the reviewer, MALDI-imaging provide a new dimension for cellular analyses but unfortunately, current MALDI IMS instruments can only achieve spatial resolutions just below 20 µm making sub-cellular analysis impossible or very coarse. Moreover, the associated MS-identification method (top-down) is still in its infancy and allows, at best, identification of only proteins with very low molecular weight without a possibility of quantification. That's why, in this paper, the authors microdissected area of interest of frozen

tissues to identify the proteome. Our approach is new because thanks to our procedure we can reach a subcellular scale and analyze structures that have been fixed and isolated in a specific way. In parallel of this manuscript, our method was patented (**WO2017009579A1**).

Furthermore the throughput claimed by the authors, isolation of 900 rosettes per hour, equates to one isolation every 4 seconds. This is to short for any form of manual QC and no automated QC is described. For example Figure 1a shows that the invadosomes can also reside below the cell body and close to the nucleus, and the invadosomes can reside close together (and thus would be merged by the "morphological closing and hole filling performed during the segmentation algorithm").

In this reviewer's opinion the authors have not demonstrated how they avoid/minimize the contribution from non-invadosome structures.

We thank the reviewer for pointing at the two mentioned papers which, indeed present some automation applied to laser microdissection. The two papers are aimed at dissecting large anatomical structures (diffuse malignant mesothelioma in the case of Großerueschkamp et al.; cortex, white matter and hippocampus in Dilillo et al. 's paper). A recent paper by Brasko et al. (Nat. Commun., vol. 9, no. 1, p. 226, 2018) presents a workflow to automatically isolate individual cells, prior to single cell proteomics. Our study therefore complements the available techniques by providing the community with capabilities to target smaller structures, namely subcellular compartments. Our approach is the first of its kind to scale down the focus, reaching an unprecedented resolution. However, we added this point and references associated in the introduction (page 5, lines 92).

As for the image processing procedure, the reviewer raises concerns about morphological closing and hole filling which may result in structures' fusion. Indeed, this event may arise. However, once segmented, the particles candidates are submitted to morphometric filtering: only particles falling into a size and circularity criterion are retained. Therefore, in case fusion occurs due to the use of morphological operators, such structures are excluded based on their shape descriptors (too big to fit). Same goes for hyper-segmentation where a single invadosome might give raise to an abnormally high number of ROIs (too small to fit the criterion). This procedure enables a first automated quality check. A second quality check is also operated: once dissection has been performed, images are acquired, that might be compared to original images used for objects' detection. Comparison of images acquired before and after dissection therefore provides an offline quality check (see new figure S2). In case mis-segmentation has been detected, the collected sample is discarded.

At the very least the authors should provide the ImageJ code so that the work may be reproduced and confirmed (it is all very good that they claim they can do it but the reader is not able to check the ImageJ code to check the accuracy of the workflow). Without adding the code, itself as supplementary information the impact of the work is greatly reduced.

In order to comply with Science Openness requirements, we now provide the reader with a link to download the full code for both the ImageJ macro and plugin (both the source code and a compiled version) (see material and methods section, page 23, line 495). Both are shared at the following link: https://github.com/fabricecordelieres/IJ_PALM_Zeiss_workflow .

The reader will find there:

- -A visual and textual description of the workflow;
- -An illustrative movie, showing the workflow in action;
- -A user manual, explaining how the macro (toolset) and plugin should be installed and used;
- -A small dataset and instructions to test the toolset offline;
- -A poster recently presented as part of the NEUBIAS 2018 conference

Statistics - while it may be argued that the low throughput of the invadosome isolation makes any statistical analysis difficult (because repeats are very long, involved experiments), the independent functional validation appears strong. In this reviewer's mind, independent validation is always superior adn should be commended.

This study has been performed to make the proof of concept of this new approach. Here, the functional validation consolidates the obtained results and demonstrates the reliability of the technical process. Accordingly, to the reviewer's comment, for future projects we planned to perform technical replicates before functional validation.

Regarding the protein quantitation dilution series - This reviewer is not convinced with this methodology - primarily because the proteome of the invadosome is much smaller than that of the NIH-3T3-Src cells used for the dilution curve - accordingly one cannot assume an equal mass spectrometric response of both proteomes.

Because of a too low concentration of proteins and too high concentration of detergents, directly measure the quantity of proteins from invadosomes sample appeared impossible with classical biochemical procedures. That's why we opted to evaluate the protein concentration with LC-MS/MS analysis using a dilution range of a total extract of NIH-3T3-Src cells. Indeed, when the dynamic range of protein concentration is different between samples, it's more a global estimation than a precise measure (similarly to the estimation of a protein concentration in comparison to a range of BSA concentration). Comparing the sum of all MS intensities is one of the classical methods to determine the stoichiometry of samples, that can be used for normalization of label free proteomic analyses (Callister Stephen J. J Proteome Res. 2006 Feb; 5(2): 277–286). That's why we choose this strategy to demonstrate enrichment in invadosomal proteins. According to the reviewer's remark we replaced the term "measure" by "estimate" (page 9 line 187)

The work is based on cell lines that exhibit large invadosome rosettes that are labeled, thus enabling their localization for LCM. However, it would be enormously of interest to the reader if the authors could expand on how this technology could be developed further to investigate the proteome of invadosomes from patient tumor cells, to investigate if the invadosome proteome changes with the invasiveness of the tumor cells.

Of course, an important aspect of this technology should be a transfer to patient tumor cells. Unfortunately, data and characterization of invadosomes *in vivo* are not sufficient to apply this methodology directly into tissues. Indeed, on fixed tissue, it is not possible for the moment to visualize invadosomes. Moreover, another important difficulty concerning those structures is that

they are dynamic and can adopt various morphologies in 3D (Di Martino et al. JCS 2016). In this idea, it is difficult to evaluate the percentage of tumor cells exhibiting invadosomes in invasive tumors. An alternative strategy is to microdissect an invading group of cells to analyze their proteome and compare data with ours to highlight invadosome components involved in this process. Currently, we started this project on invasive liver carcinoma. Sentences were added to the conclusion to clarify this point (page 19, line 398).

Reviewer #2 (Remarks to the Author):

Saltel and all have an interesting approach to address the invadosome proteome. Although they here focus on the generation mechanism of the functional proteins by local translational control tat likely regulate invadosome structure and function.

Major comments:

- Confocal images (or image stacjks) increase information in Z. DO NOT USE max, intensity projection in any of the colocalization studies. Show the Z-stacks (Suppl?)

The Z-stacks are not very informative for colocalization due to the low confocal resolution in Z. Here, we didn't use max or intensity projection, only image using a pinhole of 95.54 µm. This information has been added in the microscopy material and methods section.

- Always show the non-merged channels in B/W (Suppl).

To facilitate visualization of the different markers, we modified the figures using B/W images in the Figure 3 and new Figure S4, S5 and S7.

- Fig.1a. Unclear because lack of control (Src RNAi).

In the Fig. 1a, we added a picture of NIH-3T3 WT, without overexpression of Src. These cells don't exhibit invadosomes rosettes and consequently no degradation of the ECM can be observed. At the opposite, NIH-3T3 WT cells form actin stress fibers.

- Fig.1b. Why not show image of cells after microdissection, incl. counterstain with e.g. Hoechst?

As requested by the reviewer 1, we generated an additional supplemental figure showing cells before and after microdissection (see new figure S2).

- Fig.1c. Discuss high amount of contaminating peptides.

As reported by Hodge K *et al* , the ability of the mass spectrometers to identify increasing numbers of proteins also increased the identification of undesirable peptides (those not from the protein sample). In this study, we analyzed very small amounts of material, especially during our first tests between 100 and 10 000 rosettes collected (theoretical amounts between 0.18 and 18 ng of protein). In such condition, the intensity of the majority of proteins in the sample were below the detection threshold. In this case, the mass spectrometer spends time sequencing contaminating background in the absence of more abundant proteins. In contrary, when we dissected 40 000 rosettes (equivalent to 72ng of proteins), the percentage of identified contaminating proteins became minority (5%) as soon as we exceeded the sensitivity threshold of the mass spectrometer. This result demonstrated the relevance of isotopic labeling prior to laser micro-dissection to guarantee the specificity of protein identifications. We developed this point in the discussion (page 16, line 327-343).

- Fig.3. Should NOT BE MAX PROJECTIONS

Images of the figure 3 didn't correspond to max projection, only to confocal images representing one slice.

- Fig.3b. Use B/W images for seperate channels. Discuss why some proteins of interest are present at some invadosomes but not others. Ab specificity is questionable. Use RNAi against eEF2, Caprin to addres specificity (or CHX).

We modified images and add B/W images to separate channels and to facilitate images interpretation. Concerning eEF2 and caprin 1, in this condition cells were transfected using an eEF2-GFP and caprin1-myc constructs, respectively, and some cells were not transfected explaining why cells are not stained. RNAi was used in the Fig. 3c and 3d to address the specificity and the impact of the depletion of those proteins in invadosome formation and degradation activity.

- Fig.3c. Insets are not informative at this size.

To facilitate visualization of this figure, we realized magnification of the presented images (see Figure 3c).

- Fig.4b. CLEM is ok. Discuss lack of Z-correlation (always a problem with CLEM).

Z correlation is the trickiest aspect of correlative microscopy. First of all, it is important to understand that the angle of optical sectioning made by confocal microscopy are very difficult to reproduce (most likely impossible) when performing ultramicrotomy of a resin block. The experimentator aims to reproduce the same angle as much as possible, and this is why samples are embedded to allow similar angle sectioning. Second, thickness of EM sections are different from optical sectioning done with confocal imaging. The z-step used in confocal microscopy is roughly 1 µm, while EM sections are 70-100 nm (the thickness is controlled by the ultramicrotome and the color of the sections only). These two important criteria, in addition to sample loss that can occur when cutting a resin block, make z-correlation very difficult, and often approximate. This, nevertheless, yet allows to accurately correlate ROI between the 2 modalities.

- Fig.4. Despite proper fixation protocols, ultrastructure is hampered. Why? Discuss.

Although we agree that the ultrastructure of the sample presented in figure 4 is not fully optimal, one has to realize that such procedure is designed to allow correlative microscopy, which can affect the quality of the sample preparation. This is especially the case when looking at isolated subcellular structures such as invadopodia. The slightly reduced quality of the ultrastructure is probably due to some lipid extraction that occurred during fixation. Nevertheless, such ultrastructure quality is sufficient to grasp important intracellular organelles and compartments.

- Fig.4. How are the authors sure that the small dots are ribosomes? Quite electron dense compared to the typical ribosome appearance in EM.

When performing these experiments, we submitted the samples and micrographs to experts in Electron microscopy (members of the IGBMC Electron Microscopy platform) that assisted during

the experiments. They were the ones that claimed that these dots were free ribosomes, based on the shape, the size and the opacity to electrons. We also compared these ribosomes to ER-bound ones and could observe that they were very similar.

Minor comments:

- Homemade ImageJ tools are used: Make these public in the suppl. material.

The ImageJ macro and plugin (both the source code and a compiled version) are both shared at the following link: https://github.com/fabricecordelieres/IJ_PALM_Zeiss_workflow . This link has been added in the material and methods section (page 23, line 478_479).

- Nomenclature initiation factors is inconsistent (sometimes wrong) eIF3 (Not EIF3)

We agree with this point and the text was modified accordingly.

- line 645 : Surprising high amount of contaminating peptides. Please discuss.

As already asked by the reviewer 2 we developed this point in the discussion (page 16, line 327-343)

- too many mentions of 'we .... for the first time...'

We agree with this remark and the text was modified accordingly

- line 576: EM images were stacked. Please explain.

Our CLEM procedure is designed to allow 3D reconstruction of the region of interest (ROI), at nanoscale (Karreman et al, Plos One 2014). Thus, once cells are embedded in resin, blocks are serially sectioned to allow 3D reconstruction. Upon imaging of individual sections (thickness: 70 nm), consecutives images are aligned and ordered in Z one to another to allow 3D segmentation. Such procedures not only allow to track the same ROI but also to get a 3D volume of the ROI, previously imaged with confocal, but at nanoscale resolution.

Reviewer #3 (Remarks to the Author):

The authors employ Laser Microdissection of mRuby-LifeAct labeled fixed cell protrusion rosettes to study the proteome of the protrusions as “invadosomes”. The claimed novel finding is the authors identification of a large number of proteins involved in mRNA translation. The authors conclude that “invadasomes concentrate mRNA and exhibit their own local translation activity. A notable aspect of the manuscript is the development of automated image analysis method to reduce the considerable time of the microscopic operator to conduct the microdissection of large number of labeled cell protrusions for the MS analysis. The technical methods are exciting and presented well, and they should be of broad interest. Unfortunately the new knowledge gained about invadosome function, compared to past work, is very limited as delineated below.

This study brings new data about invadosomes like the internal translational machinery and, our work here can be useful and serve as a resource work to investigate the involvement of new proteins in invadosome formation and function. The number of new proteins identified with this work is really important, and for this study we only focused on RNA translation factors but we already tested some other elements of the enriched protein list and interestingly found that they also play a role in invadosome formation and function (data not shown).

1. The authors should make a list of the functionally important categories of known proteins known to be within invadapodia involved in matrix degradation, chemotactic sensing, actin protrusion regulation, adhesion, de adhesion and transcriptome regulation. Under each category they should list whether or not they found this molecule enriched in the invadosomes by MS compared to the non invadosome cell body. Many of the expected proteins are missing from their MS list. Typical invadosome markers well established to be enriched within invadosomes include actin-regulatory proteins such as the Arp2/3 complex, cortactin and WASP or N-WASP (Guirguis R. et al 1987, Linder and Aepfelbacher, 2003, 2009; Buccione et al., 2004, and 2009; Gimona and Buccione, 2006; Weaver, 2006; which colocalize with the actin- rich core of both structures. Functionally invadosomes are regulated by tyrosine kinase signaling, corresponding to a high local enrichment of phosphotyrosine residues (Linder and Aepfelbacher, 2003; Luxenburg et al., 2006). Invadosomes are also enriched in matrix metalloproteinases, both membrane associated and secreted latent enzymes (Journal of Cell Science 122, 3009-3013), integrins, growth factor chemotactic receptors, and focal adhesion signaling proteins such as RhoA-Rho-kinase.

In the enriched protein lists we identified several classical invadosomes markers, notably those that are cited by the reviewer, like Arp2/3 complex, cortactin and WASP. All this list is now organized in functionally categories in the new table S2 and in the new figure S3.

Functionally, invadosomes are regulated by tyrosine kinase signaling and a multitude of proteins into invadosomes are phosphorylated. In this first study, we did not focus on phosphorylated proteins, but, of course, this question is very interesting and can be part of a new exciting project.

2. The authors fail to find by mass spec (MS) many of the expected MMPs, TIMPs, Integrins, Focal Adhesion and TK proteins confirmed to be involved in the function of invadopodia. The finding of mRNA processing proteins by MS compared to these other expected proteins may be simply due to their much higher relative abundance compared to the other invadosome molecular machinery, and the low sensitivity of the MS methods used.

We agree that mRNA processing proteins seems to be very abundant in these structures. And using our strategy we were unable to identify matrix metalloproteinases, perhaps due to the difficulty to obtain peptides in mass spectrometry for this protein family. However, it could be due to the fact that even if they play a crucial role into invadosomes, they may not be abundant in comparison, for example, with actin or actin-binding proteins such as Arp 2/3 (see table S2 and figure S3). So, we are confident on our methodology and think that an increase of material accumulation combined with an increase spectrometer sensitivity will give in future more precise data about molecular invadosome composition.

3. The title is somewhat misleading since the manuscript is a proteomic based analysis of invadosomes, and not a discovery of the mRNA species that are selectively translated at the invadosome body.

We agree and we modified the titled accordingly. The new proposed title is now:

An active translation machinery controlling invadosome formation highlighted by a laser capture-proteomic combined analysis

4. Even though the authors show by several independent methods that proteins involved in mRNA translation and that RNA is localized in the invadosome they don't take this finding further to show its mechanistic role. The manuscript and does not discover novel specific mRNA species for proteins specifically involved in functional aspects of invadosome function. What specific proteins are being locally translated in the invadosome and why is this important for the invadosome function?

In the Fig. 4c, we demonstrated that actin mRNAs are enriched in invadosome structures in comparison with other actin organization as stress fibers. Interestingly, actin stress fibers are more stable and less impacted by CHX treatment in comparison with invadosomes which correlates with the importance of local translation for invadosome maintenance. The referee question is pertinent, and corresponds to the next step of this project. Indeed, we have now to reproduce the same experiment and after microdissection of the invadosomes, to realize a RNAseq analysis in order to identify mRNAs locally translated in invadosomes.

Right now, we can just propose that these huge actin-based structures need a local actin translation to maintain their ultrastructure.

5. More information should be provided describing the automation method so it can be used and reproduced by others. How will the authors make it available?

This point was asked by the reviewer 1 and 2 and now the ImageJ macro is available (see comments to reviewer 1).

Minor comment. The authors do not review invadosome or pseudopod shaving after cytokine induction of invasion as previous means to enrich invadosomes (Guirguis R. 1987 Science), or adequately review previous algorithms for pseudopod tracking Bosgraaf et al. This would round out the review of past literature.

We agree with this point and we added references to complete the introduction and the state of the art. (page 5, lines 97-100).

Reviewer #4 (Remarks to the Author):

In this study, Ezzoukhry et al, developed a pipeline that combines laser micro-dissection and mass spectrometry to analyze the molecular composition of cellular structures called invadodosmes. Besides classical invadosome markers, they found specific components of translational machinery, suggesting that invadosomes represents sites of active protein translation.

The study will be of interest to a broad community because of the unique combination of methods that allows studying molecular composition of subcellular structures. As it requires only small amount of material, it will also be a powerful method to identify protein signatures of specific regions of tissues. Besides methodological advances, the study also provides novel findings. Beautiful correlative light and electron microscopy and functional assays convincingly show that there is an active protein translation at invadosomes. Data are clear and convincing and statistical methods are appropriate.

We thank the reviewer to find our study interesting for the community.

I only have few concerns. The whole study is done using fibroblasts overexpressing Src that develop numerous and well-defined rosettes. As fibroblasts are not invasive cells per se, it is important to validate the study, or at least the major findings, in intrinsically invading cells such as cancer cells or osteoclasts. Proteomic study of cancer cells would be difficult as invadopodia are much smaller structures, but localization study of eEF1A1, eEF2 and caprin1, and eventually depletion assays, could be performed.

Indeed, normal fibroblasts are not invasive cells, however after Src activation those cells present a high number of invadosomes and became invasive (see new Fig 1a). Of course, this is an important point to validate our study in another model.

First, we confirmed the localization of the three markers used in the study, eEF1A1, eEF2 and caprin1 in linear invadosomes. NIH-3T3-Src cells were seeded into type I collagen fibrils and despite the new organization of the invadosome structures and a variation in their molecular composition, these three proteins are still enriched on those invasive structures (see new figure S5).

To go further, we tried to localize those molecules in other invadosome models, but the result is not clear, this difficulty can be due to the smaller size of those structures. But to address a potential involvement of those proteins in a tumor cell type, we knocked down eEF1A1, eEF2 and caprin1 in hepatoma cells Huh6, that are able to form invadosomes. Depletion of all three proteins impacts invadosome formation and activity in Huh6 cells (see new figure S6, for eEF1A1, eEF2; data not shown for Caprin 1). This result shows that the implication of translation-linked proteins in invadosome formation is not restricted to NIH-3T3-Src rosettes.

Minor points:

1. Fig. 1a. It would be s to show that degradation of the matrix is specifically occurring at rosettes. Thus, co-localization of invadosomes and gelatin degradation foci should be shown.

We added pictures in Fig. 1a, where we can observe a co-localization between the actin ring of the rosette and the corresponding matrix degradation.

2. Fig 3e. it would be better to split channels and presented them as black/white images in order to appreciate enrichment of eEF1A1, eEF2 and caprin1 in rosettes relative to the rest of the cell.

The Fig. 3b was modified accordingly

3. Data presented at Fig 3e should be quantified:

• enrichment of eEF1A1, eEF2 and caprin1 in rosettes relative to the rest of the cell

Using scan line representations, we demonstrate the enrichment of those markers in invadosomes in comparison to the rest of the cell (see figure below). However, this quantification is really difficult due to the heterogeneity of the rosette and the different localization of the markers such as eEF2 (see Fig R1).

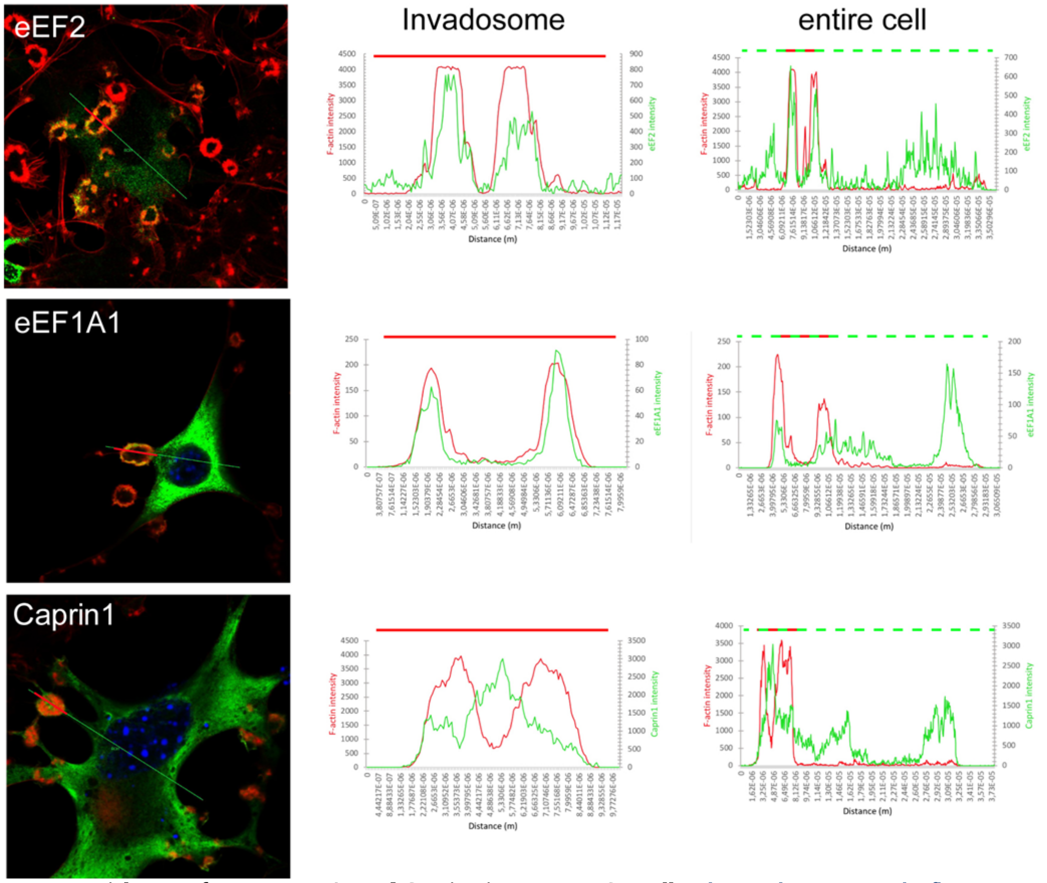

Figure 1 : Enrichment of eEF2, eEF1A1 and Caprin1 in NIH-3T3-Src cells. *The graphs represent the fluorescence intensity of F-actin (red) and the protein of interest (red) in rosettes and the entire cell along the scan line represented in the confocal images associated.*

• Proportion of rosettes containing eEF1A1, eEF2 and caprin1

A large majority of transfected cells are associated with eEF1A1, eEF2 and caprin1. In rare cases the staining is low and could be correlated with a specific maturation status of the invadosome rosette. Here, a dynamic analysis will bring more informative data.

• If eEF1A1, eEF2 and caprin1 are not present in all rosettes, it would be interesting to investigate if they are associated with newly forming or actively degrading rosettes.

Interestingly, as we showed in the movie of eEF2 during invadosome formation, the eEF2 localization and the enrichment of this protein can evolve during the formation and the stabilization of the invadosome structure (see Figure S4b). This is also the case for the protein eIF4E (see new Figure S7a). Indeed, we can notice an accumulation of this protein in the center of the invadosome rosette. Interestingly, a ring is also present and surrounds the actin. We can notice a similarity with the staining of the reticulum endoplasmic (see figure 4a). This result indicates that the localization of the translation markers may indeed vary during the invadosome formation/deformation cycle. Even if this required further investigation, this point has been added to the discussion (page18, line 378).

4. While degradation capacity of cells was reduced upon depletion caprin1, there were no effect on invadosomes formation. The authors should at least discuss what could be the underlying mechanism.

Invadosome formation and degradation can be disconnected, of course an impact on formation directly altered matrix degradation (Destaing o. et al 2014, Cell Adh Migr). But the opposite is not always true, for example MMPs inhibition depending the invadosome model can promote a destabilization of the actin structure or not. This point was added to the discussion part of the manuscript (page 17-18, lines 371-380).

Reviewers' Comments:

Reviewer #1:
Remarks to the Author:
The authors have provided a revised manuscript and addressed a number of this reviewer's concerns. However the principal issues remain. While the automated selection of fluorescently labeled features is a novel development, the dimensions are abnormally large, and cannot be translated into the rosettes found with clinical patients. Furthermore the throughput of the isolation precludes QC of the isolation (in the response to the reviewers the authors describe methods that can be applied as a QC step, but it is far from clear how much of this was actually carried out). Considering the number of concerns also raised by reviewer 4 concerning the proteins identifications, and the lack of validation (which again the authors describe as future work) means the manuscript represents an important, but small, technique development, with insufficient validation. It is the insufficient validation that makes this reviewer think the manuscript as it currently stands is not of sufficient impact for the journal. If the authors can better address the validation aspects then they should do so and resubmit. Currently the manuscript is too observational, and the scope for isolating non invadosome structures too great.

Reviewer #2:
Remarks to the Author:
The authors adequately addressed all points initially raised by myself. I would suggest to discuss the technical points raised for Fig.4 and line 576 not only in the rebuttal, but also include this information in the manuscript.

Reviewer #3:
Remarks to the Author:
The modifications are acceptable for this reviewer.
The first value of this manuscript is the method (shown as a proof concept) of using laser microdissection and proteomics to study cultured cancer cell invadosomes. The second value is the implication of functioning RNA in the invadosome, found by the technology. The functional eEF2 and eEF1A data is a needed addition to the manuscript.

Reviewer #4:
Remarks to the Author:
The authors answered to all my concerns.

Point-by-point response to the reviewer comments

REVIEWERS' COMMENTS:

Reviewer #1 (Remarks to the Author):

The authors have provided a revised manuscript and addressed a number of this reviewer's concerns. However the principal issues remain. While the automated selection of fluorescently labeled features is a novel development, the dimensions are abnormally large, and cannot be translated into the rosettes found with clinical patients.

For this study and this proof of concept, we adapted the laser microdissection and the corresponding area to the structure of interest, the invadosome rosettes. The principal aim of our study was not to use this strategy for invadopodia of clinical patients. It was to use this approach to find new markers for invadosomes and secondly test their relevance in invasion *in vitro*, and in a second step *in vivo* and finally in patients. Using this strategy, we found a new function for invadosome formation and activity and we established a list of new markers for these invasive structures including eEF2 and eEF1A1, that we validated in two different cell types (see Figure 3 and Supplementary Figure 5 and 6).
Moreover, we generated a list of proteins now available for the community to investigate their impact in cell invasion and in invadosome formation.
To conclude, we don't really understand the term "rosettes found with clinical patient", invadosomes observed after tumor cells isolation correspond to individual dots, of course to investigate this kind of structures, we have to adapt our methodology, as described in the discussion (see page 15-16, lines 471-480). Indeed, to this, we will have to realize slices of the cell to eliminate the dorsal part of the cell, and to reduce the contamination of cytoplasmic proteins. But the laser microdissection resolution is compatible with the dimension of these structures with a diameter of 1 µm for the actin core. Also, the number of invadosomes necessary to obtain the same level of identification should be more important. This work will be done first in tumor cell lines with a high percentage of cells presenting invadopodia, because based on the literature it is clear that the number of primary tumor cells exhibiting invadosomes will not be sufficient.

Furthermore the throughput of the isolation precludes QC of the isolation (in the response to the reviewers the authors describe methods that can be applied as a QC step, but it is far from clear how much of this was actually carried out).

We apologize, because we forgot to indicate that, after automatic selection of the ROI, the operator can deselect the aberrant and not specific structures. This will increase the specificity of the approach as a quality control of the selection. However, we can see on the pictures extracted from the analysis that the criteria used to select invadosomes are very strict. As other global approach all the targets identified with our strategy have to be confirmed and validated by other and complementary experiments as we performed for eEF2, eEF1A1 and the translational machinery.

Considering the number of concerns also raised by reviewer 4 concerning the proteins identifications, and the lack of validation (which again the authors describe as future work) means the manuscript represents an important, but small, technique development, with insufficient validation. It is the insufficient validation that makes this reviewer think the manuscript as it currently stands is not of sufficient impact
for the journal.

Showing additional experiments, we answered to the questions raised by the reviewer 4, especially on validation using another cellular model.

If the authors can better address the validation aspects then they should do so and resubmit. Currently the manuscript is too observational, and the scope for isolating non invadosome structures too great

Reviewer #2 (Remarks to the Author):

The authors adequately addressed all points initially raised by myself. I would suggest to discuss the technical points raised for Fig.4 and line 576 not only in the rebuttal, but also include this information in the manuscript.

As suggested, we included in the discussion the technical points raised for the Fig.4 developed in the previous rebuttal (see page 17-18 lines 523-526). And we thank the reviewer for these constructive comments.

Reviewer #3 (Remarks to the Author):

The modifications are acceptable for this reviewer.
The first value of this manuscript is the method (shown as a proof concept) of using laser microdissection and proteomics to study cultured cancer cell invadosomes. The second value is the implication of functioning RNA in the invadosome, found by the technology. The functional eEF2 and eEF1A data is a needed addition to the manuscript.

We thank the reviewer for these remarks and the additional and functional eEF2 and eEF1A data are now included in the manuscript (see Figure 3 and Supplementary Figure 5 and 6).

Reviewer #4 (Remarks to the Author):

The authors answered to all my concerns.

We thank the reviewer for the comments and the additional experiments suggested that improved the quality of our manuscript.
